# Exposures to perfluoroalkyl substances and asthma phenotypes in childhood: an investigation of the COPSAC2010 cohort

**DOI:** 10.1016/j.ebiom.2023.104699

**Published:** 2023-07-08

**Authors:** Astrid Sevelsted, Casper-Emil Tingskov Pedersen, Gözde Gürdeniz, Morten Arendt Rasmussen, Jörg Schullehner, Kalliroi Sdougkou, Jonathan W. Martin, Jessica Lasky-Su, Andreanne Morin, Carole Ober, Ann-Marie Malby Schoos, Jakob Stokholm, Klaus Bønnelykke, Bo Chawes, Hans Bisgaard

**Affiliations:** a COPSAC, Copenhagen Prospective Studies on Asthma in Childhood, Herlev and Gentofte Hospital, University of Copenhagen, Copenhagen, Denmark; b Research Unit for Environment, Work and Health, Department of Public Health, Aarhus University, Aarhus, Denmark; c Science for Life Laboratory, Department of Environmental Science, Stockholm University, Stockholm, Sweden; d Channing Division of Network Medicine, Department of Medicine, Brigham and Women's Hospital and Harvard Medical School, Boston, MA, USA; e Departments of Human Genetics, The University of Chicago, Chicago, IL, USA; f Department of Pediatrics, Slagelse Hospital, Slagelse, Denmark

**Keywords:** MeSH, PFOS, PFOA, Metabolomics, Xenobiotics, Child, Mother-child cohort, Asthma, Allergy, Sensitization, FeNO, hs-CRP

## Abstract

**Background:**

Exposure to perfluoroalkyl substances may affect offspring immune development and thereby increase risk of childhood asthma, but the underlying mechanisms and asthma phenotype affected by such exposure is unknown.

**Methods:**

In the Danish COPSAC2010 cohort of 738 unselected pregnant women and their children plasma PFOS and PFOA concentrations were semi-quantified by untargeted metabolomics analyses and calibrated using a targeted pipeline in mothers (gestation week 24 and 1 week postpartum) and children (age ½, 1½ and 6 years). We examined associations between pregnancy and childhood PFOS and PFOA exposure and childhood infections, asthma, allergic sensitization, atopic dermatitis, and lung function measures, and studied potential mechanisms by integrating data on systemic low-grade inflammation (hs-CRP), functional immune responses, and epigenetics.

**Findings:**

Higher maternal PFOS and PFOA exposure during pregnancy showed association with a non-atopic asthma phenotype by age 6, a protection against sensitization, and no association with atopic asthma or lung function, or atopic dermatitis. The effect was primarily driven by prenatal exposure. There was no association with infection proneness, low-grade inflammation, altered immune responses or epigenetic changes.

**Interpretations:**

Prenatal exposure to PFOS and PFOA, but not childhood exposure, specifically increased the risk of low prevalent non-atopic asthma, whereas there was no effect on atopic asthma, lung function, or atopic dermatitis.

**Funding:**

All funding received by COPSAC are listed on www.copsac.com. The 10.13039/501100003554Lundbeck Foundation (Grant no R16-A1694); The 10.13039/501100009708Novo Nordic Foundation (Grant nos NNF20OC0061029, NNF170C0025014, NNF180C0031764); The Ministry of Health (Grant no 903516); 10.13039/100007398Danish Council for Strategic Research (Grant no 0603-00280B); and The Capital Region Research Foundation have provided core support to the COPSAC research center. COPSAC acknowledges the National Facility for Exposomics (SciLifeLab, Sweden) for supporting calibration of the untargeted metabolomics PFAS data. BC and AS has received funding for this project from the 10.13039/501100007601European Union’s Horizon 2020 research and innovation programme (BC: grant agreement No. 946228 DEFEND; AS: grant agreement No. 864764 HEDIMED).

**Research in context**

**Evidence before this study**

Perfluoroalkyl substances are persistent and bioaccumulative exogenous chemicals in the human body with a range of suspected negative health effects. It is hypothesised that exposure during prenatal and early postnatal life might have particularly detrimental effects on the developing immune system, which could affect the risk of asthma in childhood. We did a systematic search using various combinations of the keywords: Perfluorooctane sulfonate (PFOS); perfluorooctanoic acid (PFOA); child; mother; longitudinal; asthma; sensitization; allergy.

Both PFOS and PFOA exposure have previously been investigated in relation to childhood asthma and related outcomes, and a recent meta-analysis found PFOS to increase the risk of atopic dermatitis, and PFOA to increase the risk of allergic rhinitis, but no consistent associations to asthma and no stratification on asthma phenotypes.

**Added value of this study**

In a longitudinal mother-child cohort we found prenatal exposure to PFOS and PFOA, but not childhood exposure, specifically increased the risk of non-atopic asthma, whereas there was no effect on atopic asthma, lung function, or atopic dermatitis.

**Implications of all the available evidence**

Conflicting results on effect of perfluoroalkyl substances and risk of asthma could be explained by lack of consistency in timing of exposure and phenotypic asthma definition.

## Introduction

Childhood asthma and atopic diseases are early, prevalent immune diseases with complex etiology. Diagnostic criteria for childhood asthma are complex and multiple phenotypes of asthma exists.^1^

Environmental exposure to a broader range and higher levels of xenobiotics, i.e., chemicals found in an organism that are extrinsic to the normal metabolism, has increased in recent generations^2^ with exposure from the food, water, clothing and the utensils.^3^ Perfluorooctane sulfonate (PFOS) and perfluorooctanoate (PFOA) are the most investigated of the per- and poly-fluoroalkyl substances (PFAS). These two substances are detectable in most maternal blood samples, easily pass the placental barrier,^4^^,^^5^ and hence can be found in amniotic fluid and umbilical cord blood of the newborn.^6^^,^^7^

Both PFOS and PFOA exposure have previously been investigated in relation to childhood asthma and related outcomes, and a recent meta-analysis found PFOS to increase the risk of atopic dermatitis, and PFOA to increase the risk of allergic rhinitis, but no consistent associations to asthma.^8^ However, there was no stratification by asthma phenotype, e.g., non-atopic vs. atopic asthma.^8^ This is important as childhood asthma is a complex disease with heterogeneous diagnostic procedures, e.g., a previous study found conflicting results for PFAS exposure between doctor-diagnosed asthma and self-reported asthma in the same Danish cohort.^9^ Additionally asthma can be categorized in subtypes of disease with different triggers and underlying mechanisms. The most common subtypes are atopic asthma, which is associated with atopic dermatitis, allergic rhinitis and eosinophilic airway inflammation, and non-atopic asthma, which is characterized by neutrophilic airway inflammation, recurrent wheeze triggered by airway infections and underlying decreased lung function/small airway caliber.

The maturation of the immune system in early life creates a window of susceptibility, explaining why early life environmental exposures such as PFASs are of particular interest for immune programming of later health and disease.^10^ Interestingly, a Faroese study found interactions between vaccination status and the effect of PFASs exposure on asthma status,^11^ suggesting that PFASs have the ability to modulate the immune system, especially during vulnerable periods in early life, which could have programming effects on specific subtypes of childhood asthma. Thus, PFASs exposure may increase infection proneness and risk of non-atopic asthma, which could act through susceptibility to systemic low-grade inflammation, an altered functional immune response to viruses and bacteria, and/or airway epigenetic changes.

The Copenhagen Prospective Studies of Asthma in Childhood 2010 (COPSAC2010) is a single-centre longitudinal clinical mother-child cohort with extensive phenotyping of childhood asthma and related diseases during childhood. PFOA and PFOS has been assessed longitudinally with measures in mothers in pregnancy and 1 week postpartum, and in children at ages ½, 1½ and 6 years. Thereby the COPSAC2010 cohort is a unique data source for exploratory investigations on the associations between PFASs and asthma. With this study we aim to utilize the COPSAC2010 cohort to make exploratory investigations on the association between PFOS and PFOA exposure in pregnancy and early childhood on atopic and non-atopic asthma phenotypes at age 6 defined by various comorbidities and biomarkers and prospectively collected data among COPSAC2010 participants, where underlying mechanisms are investigated by integrating data on systemic low-grade inflammation (hs-CRP), functional immune responses, and epigenetics.

## Methods

This study is part of the ongoing longitudinal population-based COPSAC2010 mother-child cohort, in which 738 women were enrolled at pregnancy week 24. Pregnant women were recruited by a monthly surveillance of reimbursement to general practitioners for the mandatory pregnancy visit. They received an invitation by posted mail to contact the clinic during 2008–2010. Exclusion criteria were gestational age above week 26; daily intake of more than 600 IU vitamin D during pregnancy; or having any endocrine, heart, or kidney disorders. Women who contacted the COPSAC clinic by phone received detailed verbal information. Those who were still interested and qualifying for the study received comprehensive study information by posted mail. Finally, the women attended the clinical research unit within pregnancy weeks 22–26 for a visit in the research clinic with detailed information and enrolment into the pregnancy cohort. The pregnant women and their children, including five twin pairs, attended 14 scheduled clinical visits plus acute care visits in the first 6 years of life. All the children are followed closely at the COPSAC research clinic and all diagnoses and treatment for asthma, allergy and atopic dermatitis follow predefined and validated algorithms. The COPSAC paediatricians act as the family practitioner for all participants avoiding the heterogeneity of diagnoses and treatments in the medical community.

All information was obtained by personal interview at clinical visits by medical doctors (MDs) and research assistants both with paediatric training. Medical, familial, environmental, and socio-economic histories were assessed by predefined questions and closed response categories and entered online into a dedicated database running an audit trail (Oracle database on a Novel SQL server). Data on sex was self-reported by the parents. Analyses included both males and females. Objective information and measurements were entered into the database, subsequently double-checked against source data, and finally locked for further editing. Quality assurance followed ‘Good Clinical Practice’ guidelines. Standard operating procedures (SOPs) were predefined for all study and database registration procedures.

The persons involved in the study participate voluntarily and after written informed consent by both parents. The participating families are informed that the study is unlikely to benefit their child and that discoveries will only benefit future generations. The analyses of the biobank are anonymized, and the results are not provided to the families.

A detailed list of clinical information and exposures is presented at the COPSAC website: http://copsac.com/available-data/.

### PFOS and PFOA assessments

Relative abundance of PFOS and PFOA were measured in the blood of pregnant woman at gestational week 24 and 1 week postpartum (i.e. reflecting late pregnancy) and in the child at ages ½, 1½ and 6 years using untargeted plasma metabolomic profiling from the HD4 platform Metabolon, Inc. (NC, USA).^12^

### Batch normalization

Batch normalization was performed to compensate for inter-day variation. Specifically, each metabolite was corrected in run-day blocks by registering the medians to equal one (1.00) and correcting each variable accordingly. Thereafter, the datasets from all the four platforms were imported into RStudio (Version 1.1, RStudio, Inc) for statistical analysis. The maternal and child age 18 months plasma samples were analyzed at a later stage compared to the child age 6 months and 6 years samples. Therefore, we used 20 replicated samples, which were analyzed with the 6 months and 6 years batch (t1) and with the maternal and child age 18 months batch (t2). For each compound we calculated a ratio between t2 and t1 for each 20 replicated sample and the median of the ratios was used as a correction factor for the maternal and child age 18 months dataset.

### Targeted quantification of PFOS and PFOA

A selection of 48 samples were subsequently quantified for PFOS and PFOA concentrations using a targeted re-analysis and calibration pipeline. Samples were selected from children having all study measurements and included samples from children with both low and high concentrations of PFOS and PFOA. The quantitative data were used to calibrate the relative measures for PFOS (sum of branched and linear) and PFOA in all the samples to generate semi-quantitative measures using a targeted method for perfluoroalkyl acids modified from Glynn et al. (2012).^13^

Briefly, an aliquot of 0.2 mL of plasma was placed in a 15 mL conical polypropylene centrifuge tube and spiked at a concentration of 1 ng/mL with 13 labelled internal standards (MPFAC-C-ES, Wellington Laboratories, Wellington Laboratories). The plasma was extracted by protein precipitation with 4 mL of acetonitrile (ACN), followed by sonication at room temperature for 10 min and centrifugation at 700×*g* for 5 min. The supernatant was transferred to a new 15 mL polypropylene tube and concentrated with nitrogen gas at 30 °C to a volume of 0.2 mL. The extract was then diluted to 1 mL to a 50:50 methanol:water solvent composition before undergoing dispersive clean-up. For the clean-up, the extract was transferred to a 1.5 mL tube containing 0.025 g of bulk graphitized carbon (Supelclean ENVI-Carb, Sigma Aldrich), that had been acidified with 50 μL of glacial acetic acid and vortexed for 10 s. The sample was then centrifuged for 10 min at 20,800×*g* and the top 0.5 mL was filtered with 0.2 μm nylon centrifuge filters (Thermo Scientific™ 750 μL Nonsterile Micro-Centrifugal Filters). An aliquot of 0.2 mL of the filtered extract was transferred to a glass auto-sampler vial for analysis.

Analysis was performed by ultra-high pressure liquid chromatography (HPLC, Ultimate 3000) coupled to a HRMS Q Exactive Orbitrap HF-X (ThermoFisher Scientific, Waltham, MA, USA) with electrospray ionization (ESI). The mass spectrometer was operated in negative ESI mode and alternated between a full MS scan (90–1000 *m*/*z*, 120,000 resolution FWHM at 200 *m*/*z*) and four MS2 data-independent acquisition (DIA) scans (30,000 resolution) with variable *m*/*z* precursor windows. A 10 μL sample volume was injected onto an ACQUITY UPLC BEH C18 analytical column (130 Å, 1.7 μm, 2.1 mm × 100 mm, Waters) equipped with a ACQUITY BEH C18 1.7 μM VANGUARD Pre-Column at 40 °C. Upstream of the injector, one ACQUITY UPLC BEH C18 analytical column (130 Å, 1.7 μm, 3 mm × 30 mm, Waters) was in place to separate instrumental background PFOS and PFOA from the analytes in the sample. A binary gradient elution was used, including (A) 2 mM ammonium acetate, and (B) 100% methanol at 0.35 mL/min.

The raw data were extracted and the peak area of the molecular ions of the analytes were integrated using Xcalibur software (Thermo Scientific, version 4.1). Quantification was performed using an external solvent-based calibration curve with the internal standard method.

When extrapolating the semi quantitative measures to target quantification values, several child PFOA measures were extrapolated to negative values. For this reason, child PFOA values are offset in analyses where log transform is used.

### Clinical outcomes

The primary outcomes of this study are asthma and allergy outcomes, whereas secondary outcomes are early life infections, and lung function measures.

• Common infections registered in daily diary cards at age 0–3 years.^1^
• Childhood asthma diagnosed from a validated algorithm that included recordings of five episodes of troublesome lung symptoms for at least 3 consecutive days in the diary within the preceding 6 months (i.e. recurrent wheeze); symptoms typical of asthma; the rescue use of inhaled beta2-agonist; and response to a 3-month course of inhaled glucocorticoids followed by relapse after the end of treatment. Remission was defined as a period of 12 months without relapse. Current asthma at age 6 was used as outcome.
• Atopic dermatitis was diagnosed prospectively until age 6 years of life according to the criteria of Hanifin and Rajka and with a clinical assessment.^14^ Age at onset and any atopic dermatitis before the age of 6 years was used as outcome.
• Sensitization to inhalant allergens by specific-IgE or skin prick test measured at age 6. For no sensitization children needed to be tested against minimum 80% of aeroallergens in both tests.^15^
• Blood eosinophils at age 6 was categorized by count below vs. above 0.3.
• Atopic and non-atopic asthma phenotypes are defined as asthma at age 6 years and aeroallergen sensitization, lifetime occurrence of atopic dermatitis and blood eosinophil count above 0.3 *versus* asthma at age 6 and never atopic dermatitis, no aeroallergen sensitization and blood eosinophil count below 0.3. We also investigated asthma with vs. without each of the components one by one.
• Severe asthma exacerbations 0–6 years were based on acute care visits or medical record checks and diagnosed if the child 1) needed oral prednisolone or high-dose inhaled corticosteroid (ICS) for acute asthma-like symptoms, 2) were hospitalized due to such.
• Lung function and airway inflammation measures at age 6 including FeNO; height and sex calibrated FVC, FEV1, FEV1/FVC ratio, MMEF, sRaw; and methacholine provocation test.^16^

### Potential mediators

Mechanisms of potential associations were sought investigating potential mediation using the following data.

• C-reactive protein (CRP) levels were determined in maternal blood at pregnancy week 24 and 1 week postpartum, and in the children at age 6 months by a high sensitivity CRP electrochemiluminescence-based assay from MesoScale Discovery (Meso Scale Discovery, Gaitherburg, MD).^17^ The lower limit of detection of hs-CRP was 0.007 ng/mL. The 1 week postpartum measurement was disregarded in analyses (mainly reflecting the birth).
• Functional immune profiling of response to viral- and bacterial-derived ligands, as well as selective T cell stimulators, totalling 186 parameters were determined from whole blood from 541 18-month-old infants.^18^ Spearman correlation to all measures of PFOS and PFOA were performed, and p-values are investigated for enrichment.
• DNA-methylation (DNAm) was assessed in respiratory (nasal) epithelium at 6 years.^19^

### Covariates

Covariates were chosen a priori as factors with association to PFOS/PFOA,^20^ including parity (first born, second born, third or higher); race (European descent, non-European descent); maternal CMPF (biomarker for fish intake),^21^ maternal pre-pregnancy BMI, asthma, age; social circumstances; urbanicity^22^; and ever detection of either PFOS or PFOA in waterworks supplying the family^23^ (see below).

### Urbanicity and detection of PFOS and PFOA in drinking water supply

Birth address of participants was obtained at interviews. Via a public database with all Danish addresses (https://dawadocs.dataforsyningen.dk/), the longitude and latitude were obtained. These were linked to other publicly available data for land use classification (https://land.copernicus.eu/pan-european/corine-land-cover).

Similarly each household address at birth was assigned to the specific waterworks it was supplied by.^23^ PFOS and PFOA in drinking water samples were measured by certified laboratories and registered in the national monitoring database Jupiter, from which they were extracted, quality-controlled and collated to “never measured for PFOS/PFOA”, “never detected above detection limit”; and “at least one sample above detection limit”. For both PFOS and PFOA, the highest ever amount measured at the respective waterworks was calculated. Most tests for PFOS and PFOA were done after 2018, so levels at birth were extrapolated.

### Sample size

Inclusion criteria was available data, participants with missing data on either exposure, outcomes or covariates were excluded from analysis. No data are imputed for main analyses. Sample size is based on feasibility and availability for all analyses. Sample sizes ranges from 241 to 668 depending on outcome.

### Statistics

PFOS and PFOA correlations were investigated with Spearman's correlation coefficient and visualized in a heatmap. For each time point a principal component analysis (PCA) was performed to capture co-variance of PFOS and PFOA. Additionally, a PCA of all time point measures was performed.

The effect of PFOS and PFOA were investigated individually for each time point, with maternal 1 week postpartum reflecting late pregnancy and breastmilk exposure. In all models, PFOS and PFOA were included as continuous non-transformed concentrations, giving effect estimates per ng/mL, except for models of principal components. For outcomes at age 6, principal components 1 and 2 (PC1 and PC2) of all measurements of PFOS and PFOA were additionally investigated. As sensitivity analyses log-linear effects are also investigated.

All analyses are adjusted for confounders listed above in regression models. Time-to-events outcomes were analyzed in Cox regression models. Binary outcomes in logistic regression models; count outcomes in negative binomial models; continuous outcomes in linear regression models. In all analyses interaction with child sex was investigated. In tables all outcomes are on their original scale, except for lung functions measures which are height and sex calibrated. As both multiple exposures and outcome measurements are correlated no multiple test corrections were performed. Mediation was investigated with the R package “mediation”.^24^ All analyses were done using R statistical software.

### Statistical analysis of DNAm and PFOS/PFOA concentrations

DNA-methylation (DNAm) was assessed in respiratory (nasal) epithelium at 6 years. We included 517 individuals in the DNAm analysis following the exclusion of subjects that did not pass quality control for the DNAm protocol (n = 45).

Of the total 866,836 probes on the Illumina Methylation EPIC array, we removed 23,172 with detection p-value of >0.01 in 90% of the samples, 18,695 located on sex chromosomes and 120,903 that overlapped a known common SNP (minor allele frequency above 5%) or mapped to multiple positions because of cross-hybridizing. In total, 703,565 CpGs passed these QC thresholds and were used in downstream analysis. Normalization was performed using the SWAN algorithm from the R package minfi (version 1.26) (10.1093/bioinformatics/btu049) and quantile normalization from the R package lumi (version 2.36) (10.1093/bioinformatics/btn224). We used M-values in analysis, as they provide better sensitivity to slight changes in methylation.

Principal component analysis was used to identify the associations with possible confounding variables on DNAm. DNA concentration, array and sampling location significantly correlated with at least one of the first ten principal components and array and sampling location were regressed out using ComBat (part of sva R package version 3.36) For more details on the data procedure see Morin et al., 2020.^19^

Latent factors were estimated after protecting for the PFOx using the CorrConf method^25^ and were included as covariates to correct for hidden unwanted variation. To assess the differently methylated CpGs (DMC) between individuals we used a linear model within the limma framework. Adjustment covariates included DNA concentration and sample age at DNAm collection and sex. DMCs were assessed using the Benjamini-Hochberg procedure (FDR) <5%.

### Imputation of missing data

As a sensitivity analysis data are imputed using the method described by van Buuren (2011) using the mice package in R.^26^

Ignoring the longitudinal aspect of data (i.e., repeated measures of PFAS concentrations), a wide data frame is imputed 20 times, excluding variables which are derived from other data (using the “impute, then transform” method). Default imputation settings are used when possible, however many of the numeric data could not converge using predictive mean matching and were therefore imputed with the cartesian method. Pooled estimates of 20 complete data analyses are presented in Table E6.

### Ethics

The clinical investigations of the children and collection of biobank materials have been approved by the local Committee on Health Research Ethics and the Danish Data Protection Agency (approval no H-B-2008-093), ensuring that all personal data are handled according to GDPR standards and Danish law. All participating parents/caregivers have provided verbal and written informed consent for the participation of their children and use of the biobank samples for metabolomics research, genetics, and other measurements in the project. Participants can withdraw that consent at any time and without any further explanation.

### Role of funders

The funding agencies did not have any role in design and conduct of the study; collection, management, and interpretation of the data; or preparation, review, or approval of the manuscript.

## Results

At pregnancy week 24 and 1 week postpartum, a total of 727 and 684 women, respectively, had a valid measurement of both PFOS and PFOA. In the children at age ½, 1½ and 6 years, 602, 606 and 513 had a measurement, respectively. Median values of serum concentrations of PFOS and PFOA are presented in Fig. E1.

**Fig. 1:**
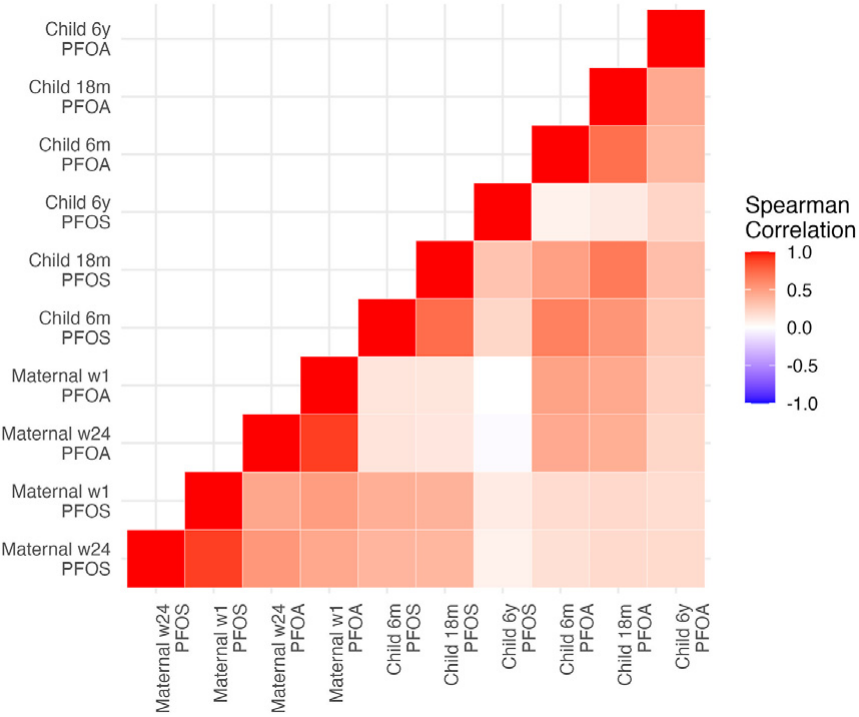
Heatmap of Spearman correlation of all PFAS measurements.

Maternal concentrations were highly correlated (Spearman R > 0.9), and child early life concentrations were similarly correlated both within child and between child and mother. At age 6 years, the child's serum concentrations were no longer strongly correlated to neither previous child measurements nor maternal concentrations (see heatmap of Spearman correlations in Fig. 1). The PCA of each time point showed that PFOS and PFOA were highly correlated, reflected in first component (PC1) explaining 75–81% in maternal and early childhood measures, and 62% of the variance in the child at age 6 year. A loading plot of a PCA of all time points is presented in online Fig. E2, showing that PC1 (47%) explains the overall correlation between concentrations of both compounds across time points, whereas PC2 (15%) explains the difference between maternal and child concentrations, regardless of compound type.

### Clinical outcomes

Cohort baseline descriptive data are presented in Table 1, and prevalences of main outcomes are presented in Table E1. Among the 437 children with data at age 6 years on both asthma, eosinophils, and inhalant sensitization, 24 children (5%) had atopic asthma, and 16 children (3%) had non-atopic asthma. Maternal serum concentrations of both PFOS and PFOA showed associations to asthma at age 6 years, driven by an association to the non-atopic asthma phenotypes with OR 1.20 pr ng/mL [1.02; 1.41], p = 0.03 and 2.81 pr ng/mL [1.22; 6.49], p = 0.02 for maternal PFOS and PFOA at week 24, respectively. These associations were stronger in log-linear models (Table E2). The increased non-atopic asthma risk was mirrored in a lower risk of any allergic sensitizations to inhalant allergens at age 6 years (Table 2). Childhood concentrations of PFOS and PFOA at age ½ and 1½, but not at age 6 years, showed a lower risk of inhalant sensitization at age 6 years. There was no association between childhood concentrations and asthma, and we found no associations to atopic dermatitis.

**Table 1:** Cohort characteristics of the 700 children in the COPSAC2010 cohort.

	N	Mean (SD)/N (%)
Sex, boys	700	360 (51.4%)
Race, European decent	700	670 (95.7%)
Parity	700	
First born		323 (46.1%)
Second born		267 (38.1%)
Third or later		110 (15.8%)
Pregnancy antibiotics	699	289 (41.3%)
Maternal asthma	700	191 (27.3%)
Maternal age	700	32 (4)
Maternal education	700	
Low		51 (7.3%)
Middle		444 (63.4%)
Higher		205 (29.3%)
Maternal pre-pregnancy BMI	695	25 (4)
Landuse	686	
Urban		307 (44.8%)
Intermediary		191 (27.8%)
Rural		188 (27.4%)
Ever detection of PFAS in waterwork	700	141 (20.1%)

**Table 2:** Clinical outcomes from exposure to PFOS and PFOA.

PFOS	Maternal w24	Maternal w1	Child 6 m	Child 18 m	Child 6 y
Time to atopic dermatitis	N = 668 HR: 1.01 [0.96; 1.06], p = 0.70	N = 658 HR: 0.99 [0.93; 1.06], p = 0.80	–	–	–
Time to asthma	N = 668 HR: 0.99 [0.93; 1.06], p = 0.80	N = 658 HR: 1.03 [0.96; 1.11], p = 0.40	N = 576 HR: 0.91 [0.84; 1.00], p = 0.04	–	–
Asthma exacerbations 0–6 yrs	N = 664 IRR: 1.00 [0.98; 1.02], p = 0.96	N = 654 IRR: 1.01 [0.98; 1.03], p = 0.62	N = 573 IRR: 1.00 [0.97; 1.02], p = 0.83	N = 582 IRR: 0.99 [0.97; 1.01], p = 0.17	–
Asthma at 6 yrs	N = 636 OR: 1.03 [0.93; 1.14], p = 0.53	N = 626 OR: 1.1 [0.98; 1.23], p = 0.11	N = 553 OR: 0.97 [0.84; 1.12], p = 0.65	N = 568 OR: 0.99 [0.9; 1.09], p = 0.87	N = 485 OR: 0.88 [0.71; 1.1], p = 0.26
Eosinophil count >0.3 at 6 yrs	N = 467 OR: 0.98 [0.92; 1.06], p = 0.65	N = 460 OR: 1 [0.92; 1.09], p = 0.99	N = 408 OR: 0.96 [0.88; 1.05], p = 0.41	N = 423 OR: 1.01 [0.95; 1.08], p = 0.72	N = 443 OR: 1.03 [0.93; 1.15], p = 0.58
Any sensitization to inhalant allergens at 6 yrs	N = 467 OR: 0.9 [0.82; 0.99], p = 0.03	N = 459 OR: 0.84 [0.73; 0.95], p=<0.01	N = 409 OR: 0.86 [0.76; 0.98], p = 0.02	N = 424 OR: 0.94 [0.86; 1.03], p = 0.19	N = 438 OR: 0.97 [0.85; 1.1], p = 0.62
Asthma at 6 yrs and inhalant sensitization	N = 464 OR: 0.78 [0.55; 1.11], p = 0.17	N = 456 OR: 0.75 [0.48; 1.16], p = 0.20	N = 406 OR: 0.8 [0.53; 1.19], p = 0.27	N = 423 OR: 0.92 [0.68; 1.25], p = 0.61	N = 436 OR: 0.93 [0.61; 1.39], p = 0.71
Asthma at 6 years and ever atopic dermatitis	N = 636 OR: 1.01 [0.85; 1.19], p = 0.94	N = 626 OR: 1.06 [0.88; 1.28], p = 0.51			
Asthma at 6 yrs and eosinophil count >0.3	N = 465 OR: 0.91 [0.72; 1.15], p = 0.44	N = 458 OR: 1 [0.78; 1.28], p = 0.99	N = 406 OR: 0.82 [0.59; 1.16], p = 0.26	N = 422 OR: 1.09 [0.91; 1.29], p = 0.35	N = 442 OR: 0.96 [0.69; 1.34], p = 0.82
Atopic asthma at 6 yrs^a^	N = 411 OR: 0.99 [0.83; 1.17], p = 0.87	N = 404 OR: 1.03 [0.85; 1.25], p = 0.77			
Asthma at 6 yrs and no inhalant sensitization	N = 464 OR: 1.15 [1.01; 1.29], p = 0.03	N = 456 OR: 1.24 [1.07; 1.42], p=<0.01	N = 406 OR: 1.07 [0.89; 1.28], p = 0.46	N = 423 OR: 1.01 [0.9; 1.14], p = 0.83	N = 436 OR: 0.91 [0.71; 1.17], p = 0.47
Asthma at 6 yrs and never atopic dermatitis	N = 636 OR: 1.05 [0.93; 1.18], p = 0.45	N = 626 OR: 1.1 [0.97; 1.26], p = 0.14			
Asthma at 6 yrs and eosinophil count ≤0.3	N = 465 OR: 1.08 [0.96; 1.22], p = 0.22	N = 458 OR: 1.14 [0.99; 1.3], p = 0.06	N = 406 OR: 1.03 [0.86; 1.23], p = 0.76	N = 422 OR: 0.93 [0.78; 1.1], p = 0.39	N = 442 OR: 0.86 [0.65; 1.13], p = 0.28
Non-atopic asthma at 6 yrs^a^	N = 411 OR: 1.2 [1.02; 1.41], p = 0.03	N = 404 OR: 1.24 [1.04; 1.48], p = 0.02			
**PFOA**	**Maternal w24**	**Maternal w1**	**Child 6 m**	**Child 18 m**	**Child 6 y**

Time to atopic dermatitis	N = 668 HR: 0.94 [0.74; 1.20], p = 0.60	N = 658 HR: 0.95 [0.70; 1.29], p = 0.70	–	–	–
Time to asthma	N = 668 HR: 0.88 [0.67; 1.17], p = 0.40	N = 658 HR: 1.02 [0.74; 1.41], p = 0.90	N = 576 HR: 0.95 [0.87; 1.03], p = 0.20	–	–
Asthma exacerbations 0–6 yrs	N = 664 IRR: 0.98 [0.9; 1.06], p = 0.56	N = 654 IRR: 0.98 [0.88; 1.09], p = 0.71	N = 573 IRR: 0.99 [0.96; 1.02], p = 0.44	N = 582 IRR: 0.99 [0.95; 1.02], p = 0.51	–
Asthma at 6 yrs	N = 636 OR: 1.09 [0.7; 1.69], p = 0.71	N = 626 OR: 1.37 [0.84; 2.24], p = 0.21	N = 553 OR: 1.02 [0.88; 1.18], p = 0.80	N = 568 OR: 1 [0.83; 1.2], p = 0.98	N = 485 OR: 0.81 [0.47; 1.42], p = 0.47
Eosinophil count >0.3 at 6 yrs	N = 467 OR: 0.89 [0.63; 1.25], p = 0.49	N = 460 OR: 0.78 [0.51; 1.19], p = 0.25	N = 408 OR: 0.97 [0.88; 1.06], p = 0.47	N = 423 OR: 0.98 [0.85; 1.13], p = 0.80	N = 443 OR: 1.25 [0.91; 1.73], p = 0.18
Any sensitization to inhalant allergens at 6 yrs	N = 467 OR: 0.93 [0.61; 1.41], p = 0.72	N = 459 OR: 0.84 [0.5; 1.43], p = 0.53	N = 409 OR: 0.97 [0.86; 1.08], p = 0.55	N = 424 OR: 0.98 [0.83; 1.16], p = 0.82	N = 438 OR: 0.99 [0.7; 1.42], p = 0.97
Asthma at 6 yrs and inhalant sensitization	N = 464 OR: 0.68 [0.18; 2.59], p = 0.57	N = 456 OR: 0.69 [0.12; 3.81], p = 0.67	N = 406 OR: 0.93 [0.67; 1.3], p = 0.69	N = 423 OR: 1.06 [0.64; 1.76], p = 0.81	N = 436 OR: 0.44 [0.11; 1.83], p = 0.26
Asthma at 6 yrs and ever atopic dermatitis	N = 636 OR: 0.77 [0.32; 1.86], p = 0.56	N = 626 OR: 0.88 [0.31; 2.49], p = 0.82			
Asthma at 6 yrs and eosinophil count >0.3	N = 465 OR: 0.43 [0.12; 1.57], p = 0.20	N = 458 OR: 0.56 [0.11; 2.77], p = 0.47	N = 406 OR: 0.95 [0.71; 1.28], p = 0.76	N = 422 OR: 1.29 [0.9; 1.86], p = 0.17	N = 442 OR: 1.04 [0.55; 1.98], p = 0.89
Atopic asthma at 6 yrs	N = 411 OR: 0.64 [0.26; 1.63], p = 0.35	N = 404 OR: 0.78 [0.26; 2.33], p = 0.65			
Asthma at 6 yrs and no inhalant sensitization	N = 464 OR: 1.53 [0.81; 2.88], p = 0.19	N = 456 OR: 2.11 [1.1; 4.05], p = 0.02	N = 406 OR: 1.08 [0.9; 1.29], p = 0.42	N = 423 OR: 1.09 [0.85; 1.4], p = 0.49	N = 436 OR: 1 [0.57; 1.76], p = 1.00
Asthma at 6 yrs and never atopic dermatitis	N = 636 OR: 1.23 [0.77; 1.98], p = 0.39	N = 626 OR: 1.55 [0.91; 2.66], p = 0.11			
Asthma at 6 yrs and eosinophil count ≤0.3	N = 465 OR: 1.7 [0.88; 3.29], p = 0.12	N = 458 OR: 2.15 [1.12; 4.15], p = 0.02	N = 406 OR: 1.01 [0.82; 1.24], p = 0.95	N = 422 OR: 0.92 [0.67; 1.25], p = 0.59	N = 442 OR: 0.64 [0.28; 1.49], p = 0.30
Non-atopic asthma at 6 yrs	N = 411 OR: 2.81 [1.22; 6.49], p = 0.02	N = 404 OR: 3.09 [1.33; 7.2], p=<0.01			

All estimates (95% CI) and z-statistic p-values present the effect per ng/mL PFOS/PFOA, in either Cox proportional hazard models or multiple logistic regression models, and are adjusted for parity, race, CMPF (biomarker for fish intake), maternal BMI, maternal asthma, social circumstances, maternal age, drinking water, and urbanicity.

a Definition of atopic and non-atopic asthma based on asthma at age 6 years and aeroallergen sensitization, atopic dermatitis (ever) and blood eosinophil count above 0.3 vs asthma at age 6 and never atopic dermatitis, no aeroallergen sensitization and blood eosinophil count below 0.3.

Combining PFOS and PFOA at each timepoint yielded similar results (Table E3). We found no evidence for sex specific effects.

PC1 scores from the combined PFAS measurements from all time points (Fig. E2), which reflected overall levels, was associated with an increased risk of non-atopic asthma at age 6 years: OR: 1.37 [1.02; 1.83], p = 0.034. PC2 scores, reflecting the shift from maternal to child, i.e., higher PC2 reflects lower maternal concentrations, was protective to the same asthma phenotype: OR: 0.51 [0.31; 0.85], p = 0.011 (Table E4).

There were no associations between either PFAS or PC scores and lung spirometry measurements at age 6, but we saw an isolated positive association between child 18 months PFOA concentration and airway resistance: estimate pr ng/mL 0.02 (SD, 0.01), p < 0.01 (Table 3). We found statistically significantly higher FeNO at age 6 by child's own concentrations of PFOS at ages 18 months and 6 years, but no associations to prenatal maternal concentrations, Table 3.

**Table 3:** Lung function at age 6 years.

PFOS	Maternal w24	Maternal w1	Child 6m	Child 18m	Child 6y
FeNO	N = 363; 0.073 (−0.2; 0.34), p = 0.60	N = 356; −0.00078 (−0.34; 0.34), p = 1.00	N = 325; 0.2 (−0.13; 0.53), p = 0.24	N = 322; 0.4 (0.15; 0.64), p=<0.01	N = 300; 0.51 (0.065; 0.96), p = 0.03
FEV1	N = 540; 0.00046 (−0.0047; 0.0056), p = 0.86	N = 531; −0.00075 (−0.007; 0.0055), p = 0.82	N = 472; −0.0028 (−0.0091; 0.0035), p = 0.38	N = 490; −0.00074 (−0.0055; 0.004), p = 0.76	N = 442; 0.0033 (−0.0053; 0.012), p = 0.45
FVC	N = 540; 0.00058 (−0.0051; 0.0062), p = 0.84	N = 531; −0.00082 (−0.0077; 0.0061), p = 0.82	N = 472; −0.0049 (−0.012; 0.0021), p = 0.17	N = 490; −0.00084 (−0.006; 0.0044), p = 0.75	N = 442; −0.00053 (−0.01; 0.009), p = 0.91
FEV1/FVC	N = 546; 3.7e-05 (−0.0018; 0.0019), p = 0.97	N = 537; 9.1e-05 (−0.0021; 0.0023), p = 0.94	N = 478; 0.0015 (−0.00073; 0.0037), p = 0.19	N = 494; 0.00016 (−0.0015; 0.0018), p = 0.85	N = 443; 0.0029 (−0.00025; 0.006), p = 0.07
Methacholine challenge test	N = 481; 0.03 (−0.13; 0.19), p = 0.71	N = 473; 0.013 (−0.18; 0.21), p = 0.89	N = 423; 0.023 (−0.17; 0.22), p = 0.82	N = 437; 0.061 (−0.09; 0.21), p = 0.43	N = 403; −0.14 (−0.4; 0.12), p = 0.30
MMEF	N = 540; −0.0054 (−0.017; 0.0065), p = 0.38	N = 531; −0.0051 (−0.02; 0.0093), p = 0.49	N = 472; −0.0024 (−0.017; 0.012), p = 0.74	N = 490; −0.0023 (−0.013; 0.0089), p = 0.69	N = 442; 0.016 (−0.0043; 0.036), p = 0.12
sRaw	N = 569; 0.00035 (−0.0073; 0.008), p = 0.93	N = 561; 0.00074 (−0.0085; 0.01), p = 0.88	N = 498; −0.00067 (−0.0099; 0.0085), p = 0.89	N = 514; 0.0028 (−0.0043; 0.01), p = 0.44	N = 471; −0.0058 (−0.018; 0.0068), p = 0.37
**PFOA**	**Maternal w24**	**Maternal w1**	**Child 6m**	**Child 18m**	**Child 6y**

FeNO	N = 363; 0.069 (−1.3; 1.4), p = 0.92	N = 356; −0.45 (−2.1; 1.2), p = 0.60	N = 325; 0.09 (−0.29; 0.47), p = 0.64	N = 322; 0.45 (−0.13; 1), p = 0.13	N = 300; 0.84 (−0.42; 2.1), p = 0.19
FEV1	N = 540; −0.018 (−0.04; 0.0041), p = 0.11	N = 531; −0.019 (−0.047; 0.0081), p = 0.17	N = 472; −0.004 (−0.011; 0.0027), p = 0.24	N = 490; −0.005 (−0.014; 0.0039), p = 0.27	N = 442; −0.018 (−0.042; 0.005), p = 0.12
FVC	N = 540; −0.018 (−0.042; 0.0056), p = 0.13	N = 531; −0.023 (−0.053; 0.0074), p = 0.14	N = 472; −0.0061 (−0.013; 0.0012), p = 0.10	N = 490; −0.0043 (−0.014; 0.0054), p = 0.39	N = 442; −0.015 (−0.041; 0.011), p = 0.26
FEV1/FVC	N = 546; −0.0019 (−0.0097; 0.0059), p = 0.64	N = 537; −0.00015 (−0.01; 0.0097), p = 0.98	N = 478; 0.0013 (−0.0011; 0.0036), p = 0.29	N = 494; −0.00079 (−0.004; 0.0024), p = 0.63	N = 443; −0.0029 (−0.011; 0.0056), p = 0.51
Methacholine challenge test	N = 481; 0.18 (−0.49; 0.84), p = 0.61	N = 473; 0.23 (−0.62; 1.1), p = 0.59	N = 423; −0.0039 (−0.22; 0.21), p = 0.97	N = 437; 0.12 (−0.16; 0.4), p = 0.40	N = 403; −0.023 (−0.8; 0.75), p = 0.95
MMEF	N = 540; −0.036 (−0.086; 0.015), p = 0.17	N = 531; −0.023 (−0.087; 0.04), p = 0.47	N = 472; −0.0026 (−0.018; 0.013), p = 0.74	N = 490; −0.012 (−0.033; 0.0092), p = 0.27	N = 442; −0.045 (−0.099; 0.0093), p = 0.11
SRaw	N = 569; 0.005 (−0.027; 0.037), p = 0.76	N = 561; 0.011 (−0.03; 0.051), p = 0.60	N = 498; 0.0078 (−0.0019; 0.018), p = 0.12	N = 514; 0.02 (0.0064; 0.033), p=<0.01	N = 471; 0.024 (−0.0097; 0.058), p = 0.16

All estimates are beta estimate (95% CI) and t-statistic p-value from generalized linear regression models with Gaussian distribution, and present change in lung function measure per ng/mL of either PFOS/PFOA, and are adjusted for parity, race, CMPF (biomarker for fish intake), maternal BMI, maternal asthma, social circumstances, maternal age, drinking water, and urbanicity. FEV1, MMEF, FVC values are calibrated for child sex and height.

Maternal and child 6 months measurement of PFOS and PFOA were not associated to increased risk of childhood infections (online Table E5).

The tendency of increased risk of non-atopic phenotypes by maternal PFAS concentrations was confirmed in imputed data, although these results were not statistically significant (Table E6).

### hs-CRP, immune responsiveness, and epigenetics

There was an isolated statistically significant cross–sectional association between maternal PFOA and maternal hs-CRP (log transformed) at pregnancy week 24, beta-coefficient 0.15 [0.02; 0.28], p = 0.028, and borderline association to PFOS 0.03 (0.01), p = 0.071, but no associations to child's hs-CRP at age 6 months (data not shown). Therefore, hs-CRP at week 24 was tested for any potential mediating effect to offspring non-atopic asthma, which was not significant: mediation effect −1.73e-05 [95% CI: −2.55e-04; 0.00], p = 0.78.

The relation between PFOS and PFOA concentrations and innate immune responsiveness towards viral and bacterial ligands and T cell stimulations were weak (|r| < ∼0.15) with none of the signals passing multiple test corrections (online Fig. E3).

Further, PFOS and PFOA concentrations during pregnancy and childhood showed no association with the changes to DNAm of nasal epithelial cells at age 6 years (online Fig. E4).

## Discussion

Repeated prenatal and early childhood serum concentrations of bio accumulative PFOS and PFOA in a Danish cohort of 738 pregnant women and their children showed that increasing prenatal exposure was associated with increased risk of a specific low prevalent non-atopic asthma phenotype by age 6 years, but not atopic asthma, asthma exacerbations, atopic dermatitis, common infections, or lung function. Paralleled to increased non-atopic asthma, prenatal PFOS was associated with a decreased risk of sensitization to inhalant allergens. These findings point towards potential prenatal programming but not a childhood effect of PFAS exposure, which was not explained by systemic low-grade inflammation, aberrant functional immune responsiveness towards viral and bacterial ligands and T cell stimulations nor epigenetic alterations.

### Strengths and limitations of the study

It is a main strength of this study that it is part of the ongoing single-center COPSAC2010 mother-child cohort study and uses meticulously collected and validated longitudinal clinical data. Thus, all data was collected at several time points and manually compared for consistency. All data was double-checked and locked, which minimized the risk of incorrect registrations in the database. The families were followed by trained staff using predefined standard operating procedures. All clinical staff and physicians had paediatric training, ensuring high quality of data collection, validation, and homogenous diagnostic procedures. Further, asthma was diagnosed following a standardized algorithm^1^ and the multiple objective assessments allowed for specified phenotype investigation.

It is a limitation that when childhood asthma is divided in phenotypes, the number of subjects investigated are very low. We cannot rule out that some associations reported are chance findings. Further it is a limitation that only PFOS and PFOA exposure was assessed. A recent report showed higher transplacental transfer of other PFAS compounds, i.e., chlorinated polyfluorinated ether sulfonates, Cl-PFESAs for PFOS and perfluorobutanoic acid, PFBA for PFOA, highlighting the need to investigate additional PFAS compounds.^27^ Furthermore, it is a limitation that the compounds were assessed through the semi-quantitative metabolomics profile, though we calibrated these values by analyzing a selected panel of samples using a targeted pipeline.^20^ Finally, although we were able to adjust the models for a range of environmental determinants of PFASs there is still a risk of residual confounding from unmeasured factors.

### Interpretation

The serum concentrations of PFOS and PFOA measured in this Danish cohort from the Zealand region are approximately three times lower than reported Faroese concentrations measured in a cohort of similar age.^11^ Childhood concentrations were also lower than a Norwegian cohort of 3-year-old's,^28^ but comparable to other European pregnancy cohorts from Denmark and Spain.^29^^,^^30^ PFOS serum concentrations decreased over the study period, with the lowest median value measured in the 6-year-old children. PFOA concentrations were higher in children than mothers, and highest among the youngest children. Serum concentrations were correlated both between compounds and timepoints. The 6-year time point was least correlated to other measurements.

The accumulation of PFOS and PFOA in the human body is tissue specific, i.e., they do not accumulate well in adipose tissues but are present in blood and highly perfused tissues, including liver, kidney and lung tissue.^31^ Some previous studies have linked maternal PFOS exposure to an increased risk of offspring asthma,^9^^,^^32^ but a recent meta-analysis did not show a consistent association between PFAS exposure and childhood asthma.^8^ There could be several explanations for these ambiguous findings. Importantly, childhood asthma is a complex disease with several subtypes and heterogeneous practices for diagnostic procedures, well reflected in the conflicting results from the same study regarding doctor-diagnosed and parent-reported asthma.^9^ Further, timing of PFAS exposure, i.e., prenatal compared to postnatal could be of importance.

The COPSAC2010 is an asthma-dedicated clinical cohort, with deep phenotyping of childhood asthma and related outcomes, which provides a very homogenous asthma diagnosis and means for subtyping asthma. Further, we included repeated measurements of PFOS and PFOA through pregnancy and early childhood. Thus, in our cohort we were able to observe an association between prenatal PFAS exposure and a specific non-atopic asthma phenotype at age 6 years, but not atopic asthma or lung function, atopic dermatitis or infection proneness. This increased risk specific for non-atopic asthma is a novel contribution to the literature of detrimental effects of PFAS exposure and may explain previous ambiguous findings with respect to asthma. Interestingly, our longitudinal exposure data further revealed that this association was specific to prenatal PFAS exposure, i.e., a suggested in utero programming, while no such association existed for childhood PFAS concentrations. Still, the results should be interpreted with caution as the analyses of the atopic and non-atopic phenotypes were limited by a low number of cases. However, we observed consistent results in auxiliary analyses exploring a range of additional atopic and non-atopic phenotypes, where there were only associations with non-atopic asthma phenotypes.

A recent NHANES study in adults found positive associations between PFAS and FeNO,^33^ which is consistent with our data showing a positive association between child concentration of PFOS and FeNO. The mechanisms behind this association are not established.^33^ FeNO is a marker of type 2 inflammation often seen in atopic phenotypes and the association between PFOS and FeNO in our data is therefore in contrast to our other findings, which are restricted to the non-atopic asthma phenotypes. Thus, these results should be interpreted with caution as the analyses also suffer from low numbers. Further, we did detect an isolated positive association between increasing child PFOA concentration at age 18 months and higher airway resistance, but this was not significant at age 6 months or cross-sectional at 6 years. This could represent a chance finding although decreased lung function/small airway caliber is often observed in children with non-atopic asthma with recurrent wheezing episodes. The findings for FeNO and airway resistance were not statistically significant for both PFOS and PFOA, which could be a power issue as the effects were small and only nominal significant but could also be caused by opposing health effects of PFOS and PFOA on these outcomes, which we previously demonstrated for childhood growth and body composition.^20^

Low-grade systemic inflammation, determined by blood hs-CRP, is elevated in pregnant women as compared to non-pregnant women.^34^ Higher pregnancy hs-CRP concentrations have been associated with smoking and dietary factors, which can act as fetal stressors.^35^^,^^36^ We found an isolated association between higher maternal PFOA concentration and hs-CRP concentration at pregnancy week 24, which could affect offspring health and disease. Thus, we speculated that the systemic low-grade inflammation at week 24 of pregnancy could play a mechanistic role in the development of non-atopic asthma, but our analysis showed no significant mediation of PFOA exposure through hs-CRP.

Finally, we did not detect any adverse effects of PFAS exposure on either immune responsiveness to bacterial and viral ligands or T cell stimulations at 18 months or the nasal epithelial DNA methylation pattern at age 6 years.

### Conclusion

The longitudinal mother-child cohort data from COPSAC2010 on prenatal and early childhood PFOS and PFOA exposure showed that maternal exposure, but not childhood exposure, specifically was associated with an increased risk of low prevalent non-atopic asthma phenotypes at age 6 years. There were no associations between PFAS exposure and risk of atopic asthma, infections, lung function, or atopic dermatitis, and no alterations in innate immune responsiveness towards viral and bacterial ligands and T cell stimulations nor epithelial DNA methylation. These findings suggest an adverse asthma subtype-specific prenatal programming effect, which adds to the list of detrimental health effects of PFAS exposure.

## Contributors

The guarantors of the study are HB and BC, from conception and design to conduct of the study and acquisition of data. AS drafted the manuscript. AS, CETP, GG, MAR performed analyses. KS and JM calibrated the semi quantitative data. JS acquired and analyzed data on drinking water. AMo and CO QCed the methylation data. AS and BC have verified the underlying data. AS, CETP, GG, MAR, JALS, AMo, CO, JS, AMMS, KB, BC, and HB have provided important intellectual input and contributed considerably to the analyses and interpretation of the data. All authors guarantee that the accuracy and integrity of any part of the work have been appropriately investigated and resolved and all have approved the final version of the manuscript. The corresponding author had full access to the data and had final responsibility for the decision to submit for publication. No honorarium, grant, or other form of payment was given to any of the authors to produce this manuscript.

## Data sharing statement

All data that supports the findings in this study, including clinical data, are available from the corresponding author upon reasonable request: participant-level personally identifiable data are protected under the Danish Data Protection Act and European Regulation 2016/679 of the European Parliament and of the Council (GDPR) that prohibit distribution even in pseudo-anonymized form, but can be made available under a data transfer agreement as a collaboration effort.

## Declaration of interests

JWM declares expert witness testimony for plaintiffs’ executive committee representing communities impacted by PFAS and AFFF contamination in the United States (re Aqueous Film-Forming Foams Products Liability Litigation, MDL 2873). AMMS was a paid speaker for ThermoFischer Scientific. All other authors declare no potential, perceived, or real conflict of interest regarding the content of this manuscript. No pharmaceutical company was involved in the study.

## References

[1] Bisgaard H., Vissing N.H., Carson C.G. (2013). Deep phenotyping of the unselected COPSAC2010 birth cohort study. Clin Exp Allergy.

[2] Diamanti-Kandarakis E., Bourguignon J.-P., Giudice L.C. (2009). Endocrine-disrupting chemicals: an Endocrine Society scientific statement. Endocr Rev.

[3] Schug T.T., Janesick A., Blumberg B., Heindel J.J. (2011). Endocrine disrupting chemicals and disease susceptibility. J Steroid Biochem Mol Biol.

[4] Bergman Å., Heindel J., Jobling S., Kidd K., Thomas Zoeller R. (2012). State-of-the-science of endocrine disrupting chemicals, 2012. Toxicol Lett.

[5] Lim X. (2019). Tainted water: the scientists tracing thousands of fluorinated chemicals in our environment. Nature.

[6] Monroy R., Morrison K., Teo K. (2008). Serum levels of perfluoroalkyl compounds in human maternal and umbilical cord blood samples. Environ Res.

[7] Stein C.R., Wolff M.S., Calafat A.M., Kato K., Engel S.M. (2012). Comparison of polyfluoroalkyl compound concentrations in maternal serum and amniotic fluid: a pilot study. Reprod Toxicol.

[8] Luo Y., Deji Z., Huang Z. (2020). Exposure to perfluoroalkyl substances and allergic outcomes in children: a systematic review and meta-analysis. Environ Res.

[9] Beck I.H., Timmermann C.A.G., Nielsen F. (2019). Association between prenatal exposure to perfluoroalkyl substances and asthma in 5-year-old children in the Odense Child Cohort. Environ Health.

[10] Prescott S.L. (2013). Early-life environmental determinants of allergic diseases and the wider pandemic of inflammatory noncommunicable diseases. J Allergy Clin Immunol.

[11] Timmermann C.A.G., Budtz-Jørgensen E., Jensen T.K. (2017). Association between perfluoroalkyl substance exposure and asthma and allergic disease in children as modified by MMR vaccination. J Immunotoxicol.

[12] Rago D., Pedersen C.-E.T., Huang M. (2021). Characteristics and mechanisms of a sphingolipid-associated childhood asthma endotype. Am J Respir Crit Care Med.

[13] Glynn A., Berger U., Bignert A. (2012). Perfluorinated alkyl acids in blood serum from primiparous women in Sweden: serial sampling during pregnancy and nursing, and temporal trends 1996-2010. Environ Sci Technol.

[14] Thorsteinsdottir S., Stokholm J., Thyssen J.P. (2019). Genetic, clinical, and environmental factors associated with persistent atopic dermatitis in childhood. JAMA Dermatol.

[15] Schoos A.-M.M., Hansen B.R., Stokholm J., Chawes B.L., Bønnelykke K., Bisgaard H. (2020). Parent-specific effects on risk of developing allergic sensitization and asthma in childhood. Clin Exp Allergy.

[16] Hallas H.W., Chawes B.L., Rasmussen M.A. (2019). Airway obstruction and bronchial reactivity from age 1 month until 13 years in children with asthma: a prospective birth cohort study. PLoS Med.

[17] Brustad N., Fink N.R., Stokholm J. (2021). Associations of 25 hydroxyvitamin D and high sensitivity C-reactive protein levels in early life. Nutrients.

[18] Thysen A.H., Waage J., Larsen J.M. (2020). Distinct immune phenotypes in infants developing asthma during childhood. Sci Transl Med.

[19] Morin A., McKennan C.G., Pedersen C.-E.T. (2020). Epigenetic landscape links upper airway microbiota in infancy with allergic rhinitis at 6 years of age. J Allergy Clin Immunol.

[20] Sevelsted A., Gürdeniz G., Rago D. (2022). Effect of perfluoroalkyl exposure in pregnancy and infancy on intrauterine and childhood growth and anthropometry. Sub study from COPSAC2010 birth cohort. eBioMedicine.

[21] Rago D., Rasmussen M.A., Lee-Sarwar K.A. (2019). Fish-oil supplementation in pregnancy, child metabolomics and asthma risk. eBioMedicine.

[22] Lehtimäki J., Thorsen J., Rasmussen M.A. (2021). Urbanized microbiota in infants, immune constitution, and later risk of atopic diseases. J Allergy Clin Immunol.

[23] Schullehner J. (2022). Danish Water Supply Areas and their links to water production facilities: an open-access data set. GEUS Bulletin.

[24] Tingley D., Yamamoto T., Hirose K., Keele L., Imai K. (2014). Mediation:RPackage for causal mediation analysis. J Stat Softw.

[25] McKennan C., Nicolae D. (2022). Estimating and accounting for unobserved covariates in high-dimensional correlated data. J Am Stat Assoc.

[26] Van Buuren S., Groothuis-Oudshoorn K. (2011). Mice: multivariate imputation by chained equations in R. J Stat Software.

[27] Cai D., Li Q.Q., Chu C. (2020). High trans-placental transfer of perfluoroalkyl substances alternatives in the matched maternal-cord blood serum: evidence from a birth cohort study. Sci Total Environ.

[28] Papadopoulou E., Sabaredzovic A., Namork E., Nygaard U.C., Granum B., Haug L.S. (2016). Exposure of Norwegian toddlers to perfluoroalkyl substances (PFAS): the association with breastfeeding and maternal PFAS concentrations. Environ Int.

[29] Jensen R.C., Andersen M.S., Larsen P.V. (2020). Prenatal exposures to perfluoroalkyl acids and associations with markers of adiposity and plasma lipids in infancy: an Odense Child Cohort study. Environ Health Perspect.

[30] Manzano-Salgado C.B., Casas M., Lopez-Espinosa M.-J. (2017). Prenatal exposure to perfluoroalkyl substances and cardiometabolic risk in children from the Spanish INMA birth cohort study. Environ Health Perspect.

[31] Pérez F., Nadal M., Navarro-Ortega A. (2013). Accumulation of perfluoroalkyl substances in human tissues. Environ Int.

[32] Dalsager L., Christensen N., Halekoh U. (2021). Exposure to perfluoroalkyl substances during fetal life and hospitalization for infectious disease in childhood: a study among 1,503 children from the Odense Child Cohort. Environ Int.

[33] Xu H., Mao Y., Hu Y., Xu B. (2021). Association between exposure to polyfluoroalkyl chemicals and increased fractional exhaled nitric oxide in adults. Environ Res.

[34] Watts D.H., Krohn M.A., Wener M.H., Eschenbach D.A. (1991). C-reactive protein in normal pregnancy. Obstet Gynecol.

[35] Abraham M., Alramadhan S., Iniguez C. (2017). A systematic review of maternal smoking during pregnancy and fetal measurements with meta-analysis. PLoS One.

[36] Nappo A., Iacoviello L., Fraterman A. (2013). High-sensitivity C-reactive protein is a predictive factor of adiposity in children: results of the identification and prevention of dietary- and lifestyle-induced health effects in children and infants (IDEFICS) study. J Am Heart Assoc.

