# Supplementary material for: Exposures to perfluoroalkyl substances and asthma phenotypes in childhood: an investigation of the COPSAC2010 cohort
Source: eBioMedicine. 2023 Jul 8;94:104699. doi: 10.1016/j.ebiom.2023.104699 (PMC10339117; doi:10.1016/j.ebiom.2023.104699)
Supplement: Supplementary Tables [file mmc1.docx]

**Table E1**

Prevalence of various atopic outcomes in the COPSAC2010 cohort

|  | **N** | **N (%) / cases (person-years)** |
| --- | --- | --- |
| Asthma at age 6 yrs | 666 | 54 (8·1%) |
| Asthma exacerbations 0-6 yrs | 695 | 147 events (4077 person-years) |
| Atopic dermatitis 0-6 yrs | 674 | 210 (31·2%) |
| Eosinophil count >0·3 at age 6 yrs | 493 | 218 (44·2%) |
| Inhalant sensitization^1^ at age 6 yrs | 493 | 117 (23·7%) |
| **Atopic asthma phenotypes** |  |  |
| Asthma and inhalant sensitization | 490 | 10 (2·0%) |
| Asthma and eosinophil count >0·3 | 491 | 16 (3·3%) |
| Asthma and ever atopic dermatitis | 666 | 21 (3·2%) |
| Atopic asthma^2^ at age 6 yrs | 437 | 22 (5·1%) |
| **Non-atopic asthma phenotypes** |  |  |
| Asthma and no inhalant sensitization | 490 | 33 (6·7%) |
| Asthma and eosinophil count <=0·3 | 491 | 26 (5·3%) |
| Asthma and never atopic dermatitis | 666 | 33 (5·0%) |
| Non-atopic asthma^2^ at age 6 yrs | 437 | 16 (3·2%) |

1: inhalant sensitization is defined as either inhalation positive skin prick test or inhalation allergen specific IgE>0.5. Only children tested for minimum 80% of inhalation allergens are included.

2: Atopic asthma defined as asthma at age 6 and either inhalant sensitization, increased Eos or ever atopic dermatitis. Non-atopic asthma defined as current asthma and neither inhalant sensitization nor increased Eos nor ever atopic dermatitis.

**Table E2**

Loglinear effects.

Clinical outcomes from exposure to PFOS and PFOA. All estimates (95% CI) and z-statistic p-values present the effect per %increase (loglinear) from either Cox proportional hazard models or multiple logistic regression model, and are adjusted for parity, race, CMPF (biomarker for fish intake), maternal BMI, maternal asthma, social circumstances, maternal age, drinking water, and urbanicity.

| **PFOS** | **Maternal w24** | **Maternal w1** | **Child 6m** | **Child 18m** | **Child 6y** |
| --- | --- | --- | --- | --- | --- |
| Time to atopic dermatitis | N=668 HR: 1·11 [0·71; 1·75], p=0·6 | N=658 HR: 0·98 [0·67; 1·45], p=0·9 | -- | -- | -- |
| Time to asthma | N=668 HR: 0·89 [0·52; 1·52], p=0·7 | N=658 HR: 1·08 [0·68; 1·72], p=0·7 | N=576 HR: 0·63 [0·4; 0·99], p=0·05 | -- | -- |
| Asthma at 6 yrs | N=636 OR: 1·34 [0·53; 3·43], p=0·54 | N=626 OR: 1·72 [0·76; 3·87], p=0·19 | N=553 OR: 0·95 [0·42; 2·14], p=0·89 | N=568 OR: 0·99 [0·5; 1·98], p=0·99 | N=485 OR: 0·63 [0·27; 1·46], p=0·28 |
| Eosinophil count >0.3 at 6 yrs | N=467 OR: 0·83 [0·45; 1·53], p=0·55 | N=460 OR: 1 [0·59; 1·67], p=0·99 | N=408 OR: 0·73 [0·43; 1·23], p=0·23 | N=423 OR: 0·93 [0·58; 1·49], p=0·76 | N=443 OR: 1·21 [0·75; 1·96], p=0·44 |
| Any sensitization to inhalant allergens at 6 yrs | N=467 OR: 0·46 [0·22; 0·97], p=0·04 | N=459 OR: 0·45 [0·24; 0·86], p=0·02 | N=409 OR: 0·46 [0·24; 0·87], p=0·02 | N=424 OR: 0·6 [0·34; 1·04], p=0·07 | N=438 OR: 0·93 [0·52; 1·65], p=0·79 |
| Asthma at 6 yrs and inhalant sensitization | N=464 OR: 0·22 [0·02; 2·02], p=0·18 | N=456 OR: 0·34 [0·05; 2·14], p=0·25 | N=406 OR: 0·39 [0·06; 2·39], p=0·31 | N=423 OR: 0·55 [0·1; 2·96], p=0·49 | N=436 OR: 0·58 [0·1; 3·2], p=0·53 |
| Asthma at 6 yrs and ever atopic dermatitis | N=636 OR: 0·94 [0·21; 4·13], p=0·93 | N=626 OR: 1·47 [0·41; 5·3], p=0·56 |  |  |  |
| Asthma at 6 yrs and eosinophil count >0.3 | N=465 OR: 0·36 [0·06; 2·22], p=0·27 | N=458 OR: 0·95 [0·21; 4·36], p=0·94 | N=406 OR: 0·43 [0·09; 2·04], p=0·29 | N=422 OR: 1·49 [0·4; 5·49], p=0·55 | N=442 OR: 0·82 [0·21; 3·25], p=0·78 |
| Atopic asthma at 6 yrs | N=411 OR: 0·77 [0·19; 3·23], p=0·73 | N=404 OR: 1·19 [0·35; 4·04], p=0·77 |  |  |  |
| Asthma at 6 yrs and no inhalant sensitization | N=464 OR: 3·8 [1·13; 12·75], p=0·03 | N=456 OR: 4·76 [1·63; 13·85], p=<0·01 | N=406 OR: 1·95 [0·66; 5·81], p=0·23 | N=423 OR: 1·48 [0·61; 3·57], p=0·38 | N=436 OR: 0·79 [0·3; 2·09], p=0·63 |
| Asthma at 6 yrs and never atopic dermatitis | N=636 OR: 1·67 [0·52; 5·35], p=0·39 | N=626 OR: 1·81 [0·67; 4·91], p=0·24 |  |  |  |
| Asthma at 6 yrs and eosinophil count <=0.3 | N=465 OR: 2·68 [0·74; 9·69], p=0·13 | N=458 OR: 2·67 [0·89; 8·02], p=0·08 | N=406 OR: 1·63 [0·51; 5·22], p=0·41 | N=422 OR: 0·76 [0·27; 2·17], p=0·61 | N=442 OR: 0·58 [0·2; 1·69], p=0·32 |
| Non-atopic asthma at 6 yrs | N=411 OR: 7·58 [1·34; 42·92], p=0·02 | N=404 OR: 5·91 [1·4; 24·95], p=0·02 |  |  |  |
| **PFOA** | **Maternal w24** | **Maternal w1** | **Child 6m** | **Child 18m** | **Child 6y** |
| Time to atopic dermatitis | N=668 HR: 0·98 [0·7; 1·37], p=0·9 | N=658 HR: 1 [0·7; 1·42], p=1 | -- | -- | -- |
| Time to asthma | N=668 HR: 0·89 [0·59; 1·33], p=0·6 | N=658 HR: 0·99 [0·65; 1·51], p=1 | N=532 HR: 0·84 [0·7; 1·01], p=0·07 | -- | -- |
| Asthma at 6 yrs | N=636 OR: 1·43 [0·7; 2·95], p=0·33 | N=626 OR: 1·74 [0·84; 3·62], p=0·14 | N=553 OR: 1·14 [0·74; 1·77], p=0·56 | N=568 OR: 1·2 [0·76; 1·91], p=0·43 | N=485 OR: 0·95 [0·45; 2·01], p=0·89 |
| Eosinophil count >0.3 at 6 yrs | N=467 OR: 0·78 [0·49; 1·24], p=0·30 | N=460 OR: 0·75 [0·46; 1·21], p=0·24 | N=408 OR: 0·9 [0·68; 1·18], p=0·43 | N=423 OR: 0·89 [0·66; 1·2], p=0·44 | N=443 OR: 1·21 [0·77; 1·88], p=0·41 |
| Any sensitization to inhalant allergens at 6 yrs | N=467 OR: 0·91 [0·53; 1·58], p=0·75 | N=459 OR: 0·86 [0·49; 1·52], p=0·60 | N=409 OR: 0·87 [0·64; 1·17], p=0·36 | N=424 OR: 0·98 [0·7; 1·39], p=0·92 | N=438 OR: 0·87 [0·53; 1·45], p=0·60 |
| Asthma at 6 yrs and inhalant sensitization | N=464 OR: 0·57 [0·12; 2·67], p=0·48 | N=456 OR: 0·58 [0·11; 3·03], p=0·52 | N=406 OR: 0·84 [0·39; 1·81], p=0·66 | N=423 OR: 1·53 [0·49; 4·85], p=0·47 | N=436 OR: 0·62 [0·2; 1·93], p=0·41 |
| Asthma at 6 yrs and ever atopic dermatitis | N=636 OR: 0·8 [0·26; 2·48], p=0·70 | N=626 OR: 1·06 [0·33; 3·45], p=0·92 |  |  |  |
| Asthma at 6 yrs and eosinophil count >0.3 | N=465 OR: 0·4 [0·11; 1·45], p=0·17 | N=458 OR: 0·51 [0·12; 2·11], p=0·35 | N=406 OR: 0·94 [0·5; 1·77], p=0·84 | N=422 OR: 1·8 [0·71; 4·54], p=0·21 | N=442 OR: 1·39 [0·42; 4·65], p=0·59 |
| Atopic asthma at 6 yrs | N=411 OR: 0·74 [0·26; 2·09], p=0·56 | N=404 OR: 0·93 [0·31; 2·8], p=0·89 |  |  |  |
| Asthma at 6 yrs and no inhalant sensitization | N=464 OR: 2·54 [0·94; 6·83], p=0·06 | N=456 OR: 3·77 [1·41; 10·13], p=<0·01 | N=406 OR: 1·66 [0·82; 3·34], p=0·16 | N=423 OR: 1·45 [0·77; 2·74], p=0·25 | N=436 OR: 1·34 [0·53; 3·38], p=0·53 |
| Asthma at 6 yrs and never atopic dermatitis | N=636 OR: 2 [0·81; 4·92], p=0·13 | N=626 OR: 2·18 [0·89; 5·36], p=0·09 |  |  |  |
| Asthma at 6 yrs and eosinophil count <=0.3 | N=465 OR: 3·41 [1·13; 10·25], p=0·03 | N=458 OR: 4·06 [1·44; 11·45], p=<0·01 | N=406 OR: 1·35 [0·64; 2·83], p=0·43 | N=422 OR: 1·05 [0·54; 2·02], p=0·89 | N=442 OR: 0·79 [0·31; 2·03], p=0·62 |
| Non-atopic asthma at 6 yrs | N=411 OR: 7·95 [1·74; 36·23], p=<0·01 | N=404 OR: 8·04 [2; 32·32], p=<0·01 |  |  |  |

*definition of atopic and non-atopic asthma based on asthma at age 6 years and aeroallergen sensitization, atopic dermatitis (ever) and blood eosinophil count above 0·3 vs asthma at age 6 and never atopic dermatitis, no aeroallergen sensitization and blood eosinophil count below 0·3.

**Table E3**

Atopic outcomes from exposure to PC1 of PCA of PFOS and PFOA. All estimates (95% CI) and z-statistic p-values present the effect per standard deviation of principal component 1 in either Cox proportional hazard models or multiple logistic regression models, and are adjusted for parity, race, CMPF (bio marker for fish intake), maternal BMI, maternal asthma, social circumstances, maternal age, landcover.

| **PFOS and PFOA, PC1** | **Maternal w24** | **Maternal w1** | **Child 6m** | **Child 18m** | **Child 6y** |
| --- | --- | --- | --- | --- | --- |
| Time to atopic dermatitis | N=668 HR: 1 [0·87; 1·14], p=1 | N=658 HR: 0·98 [0·86; 1·11], p=0·7 |  |  |  |
| Time to asthma | N=668 HR: 0·95 [0·81; 1·11], p=0·50 | N=658 HR: 1·04 [0·9; 1·2], p=0·60 | N=576 HR: 0·85 [0·72; 1·01], p=0·06 |  |  |
| Asthma exacerbations 0-6 yrs | N=664 IRR: 0·96 [0·76; 1·2], p=0·71 | N=654 IRR: 1·01 [0·82; 1·25], p=0·92 | N=573 IRR: 0·94 [0·73; 1·2], p=0·59 |  |  |
| Asthma at 6 yrs | N=636 OR: 1·08 [0·84; 1·4], p=0·54 | N=626 OR: 1·2 [0·97; 1·49], p=0·10 | N=553 OR: 0·98 [0·74; 1·31], p=0·90 | N=568 OR: 0·98 [0·75; 1·29], p=0·91 | N=485 OR: 0·8 [0·56; 1·15], p=0·23 |
| Eosinophil count >0·3 at 6 yrs | N=467 OR: 0·94 [0·79; 1·13], p=0·53 | N=460 OR: 0·95 [0·8; 1·12], p=0·54 | N=408 OR: 0·92 [0·77; 1·11], p=0·38 | N=423 OR: 1·01 [0·83; 1·23], p=0·93 | N=443 OR: 1·12 [0·93; 1·35], p=0·23 |
| Any sensitization to inhalant allergens at 6 yrs | N=467 OR: 0·83 [0·66; 1·05], p=0·11 | N=459 OR: 0·78 [0·61; 1], p=0·05 | N=409 OR: 0·82 [0·65; 1·04], p=0·10 | N=424 OR: 0·9 [0·71; 1·13], p=0·35 | N=438 OR: 0·96 [0·77; 1·2], p=0·73 |
| Asthma at 6 yrs and inhalant sensitization | N=464 OR: 0·63 [0·28; 1·38], p=0·25 | N=456 OR: 0·66 [0·3; 1·46], p=0·31 | N=406 OR: 0·74 [0·36; 1·49], p=0·40 | N=423 OR: 0·93 [0·44; 1·97], p=0·85 | N=436 OR: 0·69 [0·31; 1·51], p=0·35 |
| Asthma at 6 yrs and ever atopic dermatitis | N=636 OR: 0·94 [0·61; 1·47], p=0·80 | N=626 OR: 1·06 [0·72; 1·56], p=0·78 |  |  |  |
| Asthma at 6 yrs and eosinophil count >0·3 | N=465 OR: 0·7 [0·38; 1·31], p=0·26 | N=458 OR: 0·9 [0·52; 1·57], p=0·72 | N=406 OR: 0·78 [0·42; 1·43], p=0·42 | N=422 OR: 1·39 [0·84; 2·32], p=0·20 | N=442 OR: 0·99 [0·62; 1·6], p=0·98 |
| Atopic asthma at 6 yrs | N=411 OR: 0·87 [0·55; 1·39], p=0·57 | N=404 OR: 0·99 [0·66; 1·49], p=0·96 |  |  |  |
| Asthma at 6 yrs and no inhalant sensitization | N=464 OR: 1·41 [1·02; 1·95], p=0·04 | N=456 OR: 1·51 [1·13; 2·02], p=<0·01 | N=406 OR: 1·18 [0·82; 1·71], p=0·38 | N=423 OR: 1·09 [0·77; 1·53], p=0·63 | N=436 OR: 0·91 [0·62; 1·35], p=0·65 |
| Asthma at 6 yrs and never atopic dermatitis | N=636 OR: 1·16 [0·86; 1·56], p=0·34 | N=626 OR: 1·24 [0·97; 1·59], p=0·08 |  |  |  |
| Asthma at 6 yrs and eosinophil count <=0·3 | N=465 OR: 1·29 [0·94; 1·77], p=0·12 | N=458 OR: 1·34 [1·04; 1·74], p=0·03 | N=406 OR: 1·04 [0·71; 1·54], p=0·83 | N=422 OR: 0·83 [0·53; 1·31], p=0·42 | N=442 OR: 0·71 [0·43; 1·16], p=0·17 |
| Non-atopic asthma at 6 yrs | N=411 OR: 1·8 [1·16; 2·78], p=<0·01 | N=404 OR: 1·65 [1·12; 2·44], p=0·01 |  |  |  |

**Table E4**

Atopic and lung function outcomes from PC1 and PC2 of PCA of PFOS and PFOA (see **Figure E2**). All estimates (95% CI) and z-statistic/t-statistic p-values are from generalized regression models, logistic for odds ratios and linear for beta estimates, and are adjusted for parity, race, CMPF (bio marker for fish intake), maternal BMI, maternal asthma, social circumstances, maternal age, drinking water, landcover.

| **PFOS and PFOA** | **PC1 (overall level)** | **PC2 (maternal to child)** |
| --- | --- | --- |
| Asthma at 6 yrs | N=385 OR: 1·12 [0·93; 1·36], p=0·23 | N=385 OR: 0·74 [0·54; 1·01], p=0·06 |
| Eosinophil count >0.3 at 6yrs | N=351 OR: 0·96 [0·85; 1·09], p=0·55 | N=351 OR: 1·01 [0·84; 1·22], p=0·92 |
| Any sensitization to inhalant allergens at 6 yrs | N=344 OR: 0·92 [0·77; 1·09], p=0·31 | N=344 OR: 1 [0·8; 1·25], p=0·99 |
| Asthma at 6 yrs and inhalant sensitization | N=344 OR: 0·85 [0·48; 1·51], p=0·58 | N=344 OR: 0·91 [0·46; 1·81], p=0·8 |
| Asthma at 6 and ever atopic dermatitis | N=385 OR: 1 [0·71; 1·41], p=1 | N=385 OR: 0·81 [0·51; 1·3], p=0·39 |
| Asthma at 6 yrs and eosinophil count >0·3 | N=351 OR: 1·01 [0·69; 1·47], p=0·95 | N=351 OR: 1·05 [0·62; 1·78], p=0·85 |
| Atopic asthma at 6 yrs | N=314 OR: 1·02 [0·75; 1·39], p=0·88 | N=314 OR: 0·94 [0·61; 1·44], p=0·77 |
| Asthma at 6 yrs and no inhalant sensitization | N=344 OR: 1·28 [1·02; 1·6], p=0·033 | N=344 OR: 0·68 [0·47; 0·98], p=0·037 |
| Asthma at 6 yrs and never atopic dermatitis | N=385 OR: 1·19 [0·95; 1·48], p=0·13 | N=385 OR: 0·71 [0·47; 1·06], p=0·097 |
| Asthma at 6 yrs and eosinophil count <=0·3 | N=351 OR: 1·15 [0·93; 1·43], p=0·2 | N=351 OR: 0·62 [0·42; 0·92], p=0·017 |
| Non-atopic asthma at 6 yrs | N=314 OR: 1·37 [1·02; 1·83], p=0·034 | N=314 OR: 0·51 [0·31; 0·85], p=0·011 |
| FeNO | N=241 beta: 0·47 [-0·071; 1], p=0·09 | N=241 beta: 0·89 [0·069; 1·7], p=0·035 |
| FEV1 | N=351 beta: -0·0057 [-0·015; 0·0037], p=0·24 | N=351 beta: -0·0042 [-0·019; 0·01], p=0·57 |
| FVC | N=351 beta: -0·0045 [-0·015; 0·0058], p=0·39 | N=351 beta: -0·0056 [-0·022; 0·01], p=0·5 |
| FEV1/FVC | N=351 beta: -0·0012 [-0·0045; 0·0022], p=0·5 | N=351 beta: 0·0013 [-0·0039; 0·0066], p=0·61 |
| Methacholine | N=323 beta: 0·21 [-0·11; 0·53], p=0·2 | N=323 beta: -0·27 [-0·77; 0·22], p=0·28 |
| MMEF | N=351 beta: -0·019 [-0·041; 0·003], p=0·091 | N=351 beta: -0·0029 [-0·037; 0·031], p=0·87 |
| sRaw | N=374 beta: 0·012 [-0·0028; 0·026], p=0·12 | N=374 beta: 0·0035 [-0·019; 0·026], p=0·76 |

**Table E5**

Associations between serum concentrations of maternal and child PFOS and PFOA versus child infectious episodes in early life 0-3 years of age, tallying 1767 person-years of observation in 634 children. Estimates (95% CI) and Wald-test p-values are calculated with negative binomial regression offsetting log of persontime of observation, thereby estimating incidence rate ratios of number of infections per ng/mL of each respective PFAS, and all estimates are adjusted for parity, race, CMPF (bio marker for fish intake), maternal BMI, maternal asthma, social circumstances, maternal age, drinking water, landcover

| **perfluorooctanesulfonate (PFOS)** | **Sum of infections / children with minimum one infection (% of all)** | **Maternal w24** | **Maternal w1** | **Child 6m** |
| --- | --- | --- | --- | --- |
| Troublesome lower airway episodes | 4283 / 562 (88%) | N=634 IRR: 0·99 [0·97; 1·02], p=0·73 | N=624 IRR: 1·01 [0·98; 1·05], p=0·45 | N=550 IRR: 0·98 [0·95; 1·02], p=0·3 |
| Gastric episodes | 1124 / 474 (75%) | N=634 IRR: 1 [0·97; 1·03], p=0·91 | N=624 IRR: 1·03 [0·99; 1·06], p=0·13 | N=550 IRR: 1 [0·96; 1·03], p=0·91 |
| Lower respiratory infections | 364 / 214 (34%) | N=634 IRR: 1·01 [0·96; 1·07], p=0·57 | N=624 IRR: 1·05 [0·99; 1·11], p=0·13 | N=550 IRR: 0·99 [0·93; 1·05], p=0·7 |
| Bluespray episodes | 2199 / 347 (55%) | N=634 IRR: 0·99 [0·94; 1·04], p=0·78 | N=624 IRR: 1·01 [0·95; 1·08], p=0·66 | N=550 IRR: 0·97 [0·91; 1·03], p=0·31 |
| Colds | 7735 / 622 (98%) | N=634 IRR: 0·99 [0·97; 1], p=0·12 | N=624 IRR: 1 [0·97; 1·02], p=0·67 | N=550 IRR: 1 [0·98; 1·02], p=0·95 |
| Fever episodes | 3522 / 591 (93%) | N=634 IRR: 1 [0·98; 1·02], p=0·66 | N=624 IRR: 1·01 [0·99; 1·03], p=0·42 | N=550 IRR: 1 [0·97; 1·02], p=0·82 |
| Acute otitis media episodes | 1014 / 378 (60%) | N=634 IRR: 0·98 [0·94; 1·02], p=0·27 | N=624 IRR: 0·98 [0·94; 1·03], p=0·54 | N=550 IRR: 0·99 [0·94; 1·04], p=0·63 |
| Tonsillitis episodes | 244 / 159 (25%) | N=634 IRR: 0·93 [0·86; 0·99], p=0·027 | N=624 IRR: 0·94 [0·86; 1·02], p=0·14 | N=550 IRR: 0·97 [0·89; 1·04], p=0·39 |
| Pseudocroup episodes | 115 / 89 (14%) | N=634 IRR: 0·99 [0·91; 1·07], p=0·78 | N=624 IRR: 1·01 [0·91; 1·11], p=0·87 | N=550 IRR: 0·98 [0·89; 1·08], p=0·71 |
| Infections | 2861 / 585 (92%) | N=634 IRR: 0·99 [0·97; 1·01], p=0·33 | N=624 IRR: 1·01 [0·98; 1·04], p=0·56 | N=550 IRR: 0·99 [0·96; 1·02], p=0·44 |
| **perfluorooctanoate (PFOA)** |  | **Maternal w24** | **Maternal w1** | **Child 6m** |
| Troublesome lower airway episodes | 4283 / 562 (88%) | N=634 IRR: 0·94 [0·83; 1·07], p=0·36 | N=624 IRR: 1·02 [0·87; 1·19], p=0·81 | N=550 IRR: 0·98 [0·94; 1·02], p=0·26 |
| Gastric episodes | 1124 / 474 (75%) | N=634 IRR: 0·98 [0·86; 1·11], p=0·74 | N=624 IRR: 1·08 [0·93; 1·26], p=0·32 | N=550 IRR: 1 [0·96; 1·04], p=0·94 |
| Lower respiratory infections | 364 / 214 (34%) | N=634 IRR: 1·01 [0·81; 1·26], p=0·92 | N=624 IRR: 1·11 [0·84; 1·45], p=0·46 | N=550 IRR: 1 [0·93; 1·07], p=0·94 |
| Bluespray episodes | 2199 / 347 (55%) | N=634 IRR: 0·89 [0·72; 1·12], p=0·32 | N=624 IRR: 0·99 [0·75; 1·3], p=0·93 | N=550 IRR: 0·96 [0·9; 1·03], p=0·26 |
| Colds | 7735 / 622 (98%) | N=634 IRR: 0·97 [0·9; 1·06], p=0·51 | N=624 IRR: 1 [0·91; 1·11], p=0·96 | N=550 IRR: 1 [0·98; 1·03], p=0·8 |
| Fever episodes | 3522 / 591 (93%) | N=634 IRR: 0·95 [0·87; 1·04], p=0·27 | N=624 IRR: 0·94 [0·84; 1·05], p=0·31 | N=550 IRR: 0·98 [0·95; 1·01], p=0·14 |
| Acute otitis media episodes | 1014 / 378 (60%) | N=634 IRR: 1·01 [0·86; 1·19], p=0·87 | N=624 IRR: 1·08 [0·88; 1·33], p=0·47 | N=550 IRR: 1 [0·95; 1·05], p=0·95 |
| Tonsillitis episodes | 244 / 159 (25%) | N=634 IRR: 0·97 [0·74; 1·27], p=0·84 | N=624 IRR: 1·11 [0·8; 1·53], p=0·54 | N=550 IRR: 1·01 [0·94; 1·1], p=0·75 |
| Pseudocroup episodes | 115 / 89 (14%) | N=634 IRR: 0·94 [0·66; 1·34], p=0·73 | N=624 IRR: 0·99 [0·64; 1·52], p=0·96 | N=550 IRR: 0·92 [0·83; 1·03], p=0·14 |
| Infections | 2861 / 585 (92%) | N=634 IRR: 0·99 [0·9; 1·1], p=0·89 | N=624 IRR: 1·08 [0·95; 1·22], p=0·22 | N=550 IRR: 1 [0·97; 1·03], p=0·79 |

**Table E6**

Pooled estimates from multiple (20) imputations on the clinical outcomes with notable amounts of missing data. Table equivalent to Table 2.

| **PFOS** | **Maternal w24** | **Maternal w1** | **Child 6m** | **Child 18m** | **Child 6y** |
| --- | --- | --- | --- | --- | --- |
| Asthma at 6 yrs | OR=1·01 [0·91; 1·11], p=0·91 | OR=1·05 [0·94; 1·17], p=0·37 | OR=0·98 [0·87; 1·11], p=0·78 | OR=1 [0·92; 1·1], p=0·97 | OR=0·95 [0·78; 1·15], p=0·60 |
| Eosinophil count >0.3 at 6yrs | OR=0·98 [0·92; 1·04], p=0·50 | OR=0·99 [0·91; 1·07], p=0·72 | OR=0·95 [0·88; 1·03], p=0·22 | OR=0·98 [0·93; 1·04], p=0·59 | OR=1 [0·91; 1·1], p=0·99 |
| Any sensitization to inhalant allergens at 6 yrs | OR=0·94 [0·86; 1·03], p=0·17 | OR=0·93 [0·84; 1·04], p=0·19 | OR=0·94 [0·85; 1·04], p=0·23 | OR=0·98 [0·91; 1·06], p=0·64 | OR=1·02 [0·92; 1·13], p=0·70 |
| Asthma at 6 yrs and inhalant sensitization | OR=0·81 [0·62; 1·05], p=0·11 | OR=0·8 [0·58; 1·1], p=0·17 | OR=0·81 [0·6; 1·11], p=0·20 | OR=0·94 [0·72; 1·21], p=0·61 | OR=0·89 [0·58; 1·35], p=0·57 |
| Asthma at 6 yrs and ever atopic dermatitis | OR=1 [0·85; 1·17], p=0·97 | OR=1·02 [0·85; 1·23], p=0·81 |  |  |  |
| Asthma at 6 yrs and eosinophil count >0.3 | OR=0·94 [0·78; 1·12], p=0·47 | OR=0·96 [0·78; 1·18], p=0·70 | OR=0·9 [0·71; 1·14], p=0·39 | OR=1·03 [0·9; 1·18], p=0·66 | OR=0·98 [0·75; 1·28], p=0·90 |
| Atopic asthma at 6 yrs | OR=0·94 [0·82; 1·08], p=0·38 | OR=0·96 [0·81; 1·13], p=0·60 |  |  |  |
| Asthma at 6 yrs and no inhalant sensitization | OR=1·06 [0·95; 1·18], p=0·27 | OR=1·11 [0·99; 1·25], p=0·08 | OR=1·04 [0·91; 1·18], p=0·60 | OR=1·01 [0·91; 1·13], p=0·79 | OR=0·97 [0·78; 1·2], p=0·79 |
| Asthma at 6 yrs and never atopic dermatitis | OR=1·01 [0·89; 1·14], p=0·88 | OR=1·06 [0·93; 1·21], p=0·40 |  |  |  |
| Asthma at 6 yrs and eosinophil count <=0.3 | OR=1·04 [0·92; 1·16], p=0·55 | OR=1·09 [0·96; 1·24], p=0·19 | OR=1·03 [0·89; 1·19], p=0·74 | OR=0·97 [0·84; 1·12], p=0·69 | OR=0·93 [0·71; 1·21], p=0·58 |
| Non-atopic asthma at 6 yrs | OR=1·08 [0·95; 1·23], p=0·24 | OR=1·14 [1; 1·31], p=0·06 |  |  |  |
| **PFOA** | **Maternal w24** | **Maternal w1** | **Child 6m** | **Child 18m** | **Child 6y** |
| Asthma at 6 yrs | OR=0·99 [0·64; 1·53], p=0·97 | OR=1·22 [0·75; 2], p=0·42 | OR=0·99 [0·88; 1·12], p=0·88 | OR=0·97 [0·81; 1·15], p=0·70 | OR=0·81 [0·5; 1·33], p=0·40 |
| Eosinophil count >0.3 at 6yrs | OR=0·89 [0·65; 1·23], p=0·49 | OR=0·83 [0·55; 1·25], p=0·37 | OR=0·96 [0·89; 1·03], p=0·27 | OR=0·97 [0·86; 1·08], p=0·56 | OR=1·13 [0·86; 1·48], p=0·39 |
| Any sensitization to inhalant allergens at 6 yrs | OR=1·35 [1·01; 1·79], p=0·04 | OR=1·39 [0·95; 2·03], p=0·09 | OR=1·03 [0·95; 1·13], p=0·45 | OR=1·06 [0·95; 1·18], p=0·26 | OR=1·05 [0·78; 1·41], p=0·75 |
| Asthma at 6 yrs and inhalant sensitization | OR=0·9 [0·36; 2·23], p=0·82 | OR=0·91 [0·29; 2·84], p=0·88 | OR=0·96 [0·75; 1·22], p=0·72 | OR=0·93 [0·66; 1·32], p=0·70 | OR=0·45 [0·12; 1·64], p=0·22 |
| Asthma at 6 yrs and ever atopic dermatitis | OR=0·81 [0·36; 1·81], p=0·61 | OR=0·82 [0·3; 2·22], p=0·69 |  |  |  |
| Asthma at 6 yrs and eosinophil count >0.3 | OR=0·73 [0·3; 1·8], p=0·49 | OR=0·74 [0·25; 2·2], p=0·58 | OR=0·96 [0·77; 1·19], p=0·71 | OR=1·05 [0·85; 1·29], p=0·66 | OR=0·95 [0·52; 1·73], p=0·86 |
| Atopic asthma at 6 yrs | OR=0·81 [0·42; 1·55], p=0·52 | OR=0·82 [0·37; 1·82], p=0·63 |  |  |  |
| Asthma at 6 yrs and no inhalant sensitization | OR=1·02 [0·62; 1·66], p=0·95 | OR=1·31 [0·76; 2·26], p=0·34 | OR=1 [0·87; 1·16], p=0·96 | OR=0·98 [0·81; 1·18], p=0·81 | OR=0·96 [0·57; 1·6], p=0·87 |
| Asthma at 6 yrs and never atopic dermatitis | OR=1·08 [0·66; 1·77], p=0·77 | OR=1·4 [0·81; 2·43], p=0·22 |  |  |  |
| Asthma at 6 yrs and eosinophil count <=0.3 | OR=1·1 [0·67; 1·8], p=0·71 | OR=1·44 [0·83; 2·49], p=0·19 | OR=1 [0·86; 1·17], p=0·98 | OR=0·89 [0·69; 1·15], p=0·37 | OR=0·7 [0·35; 1·42], p=0·32 |
| Non-atopic asthma at 6 yrs | OR=1·2 [0·7; 2·05], p=0·51 | OR=1·67 [0·92; 3·03], p=0·09 |  |  |  |

*definition of atopic and non-atopic asthma based on asthma at age 6 years and aeroallergen sensitization, atopic dermatitis (ever) and blood eosinophil count above 0·3 vs asthma at age 6 and never atopic dermatitis, no aeroallergen sensitization and blood eosinophil count below 0·3.
