# Supplementary material for: Exposures to perfluoroalkyl substances and asthma phenotypes in childhood: an investigation of the COPSAC2010 cohort
Source: eBioMedicine. 2023 Jul 8;94:104699. doi: 10.1016/j.ebiom.2023.104699 (PMC10339117; doi:10.1016/j.ebiom.2023.104699)

**Figure E1**

Concentrations of PFOS and PFOA by time point of measurement; maternal gestation week 24 N=686; maternal week 1 post partum N=683; child 6 months N=599; child 18 months N=601; and child 6 years N=508.

**
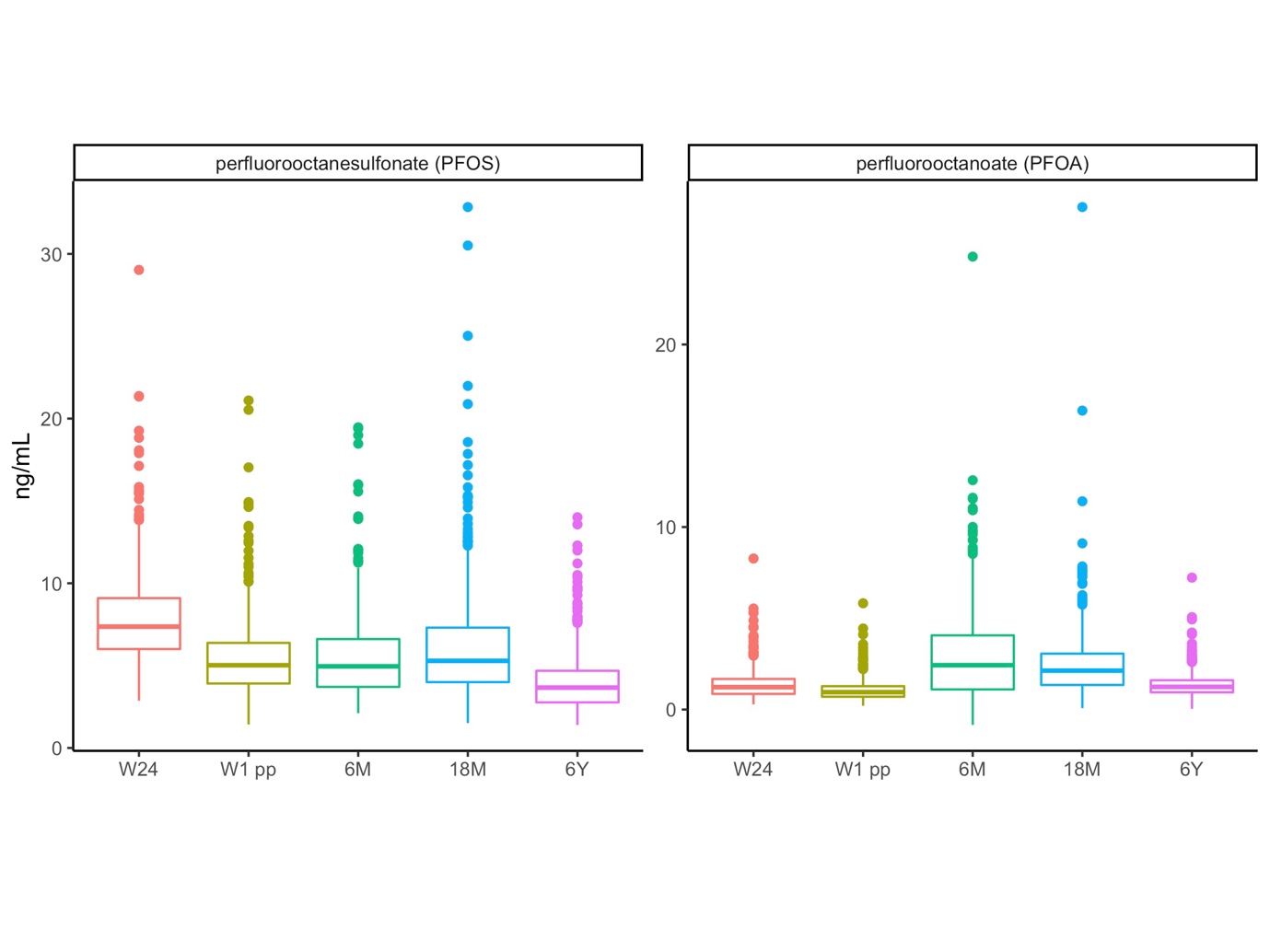
**

**Figure E2**

PCA loadings plot of all measurements of both PFOS and PFOA across all timepoints.

**
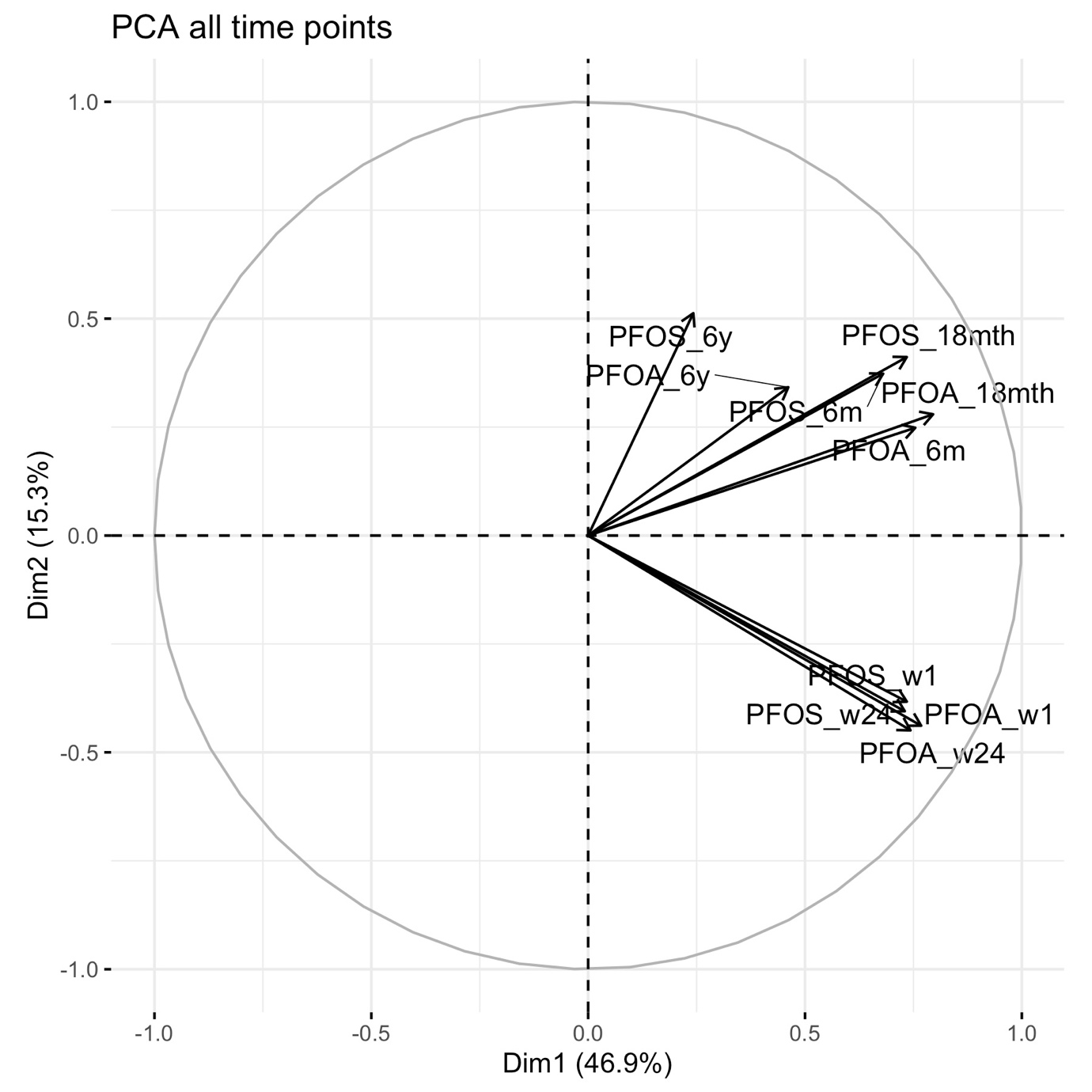
**

**Figure E3**

Immune stimulation in 18 month peripheral blood. 21 cytokines with 7 different stimulations giving 147 correlations to each measurement of maternal and child PFOA and PFOS. QQ plot of Spearman rank p-values between PFAS concentrations at four time points and the 147 immune stimulations show no inflation. No associations pass FDR.


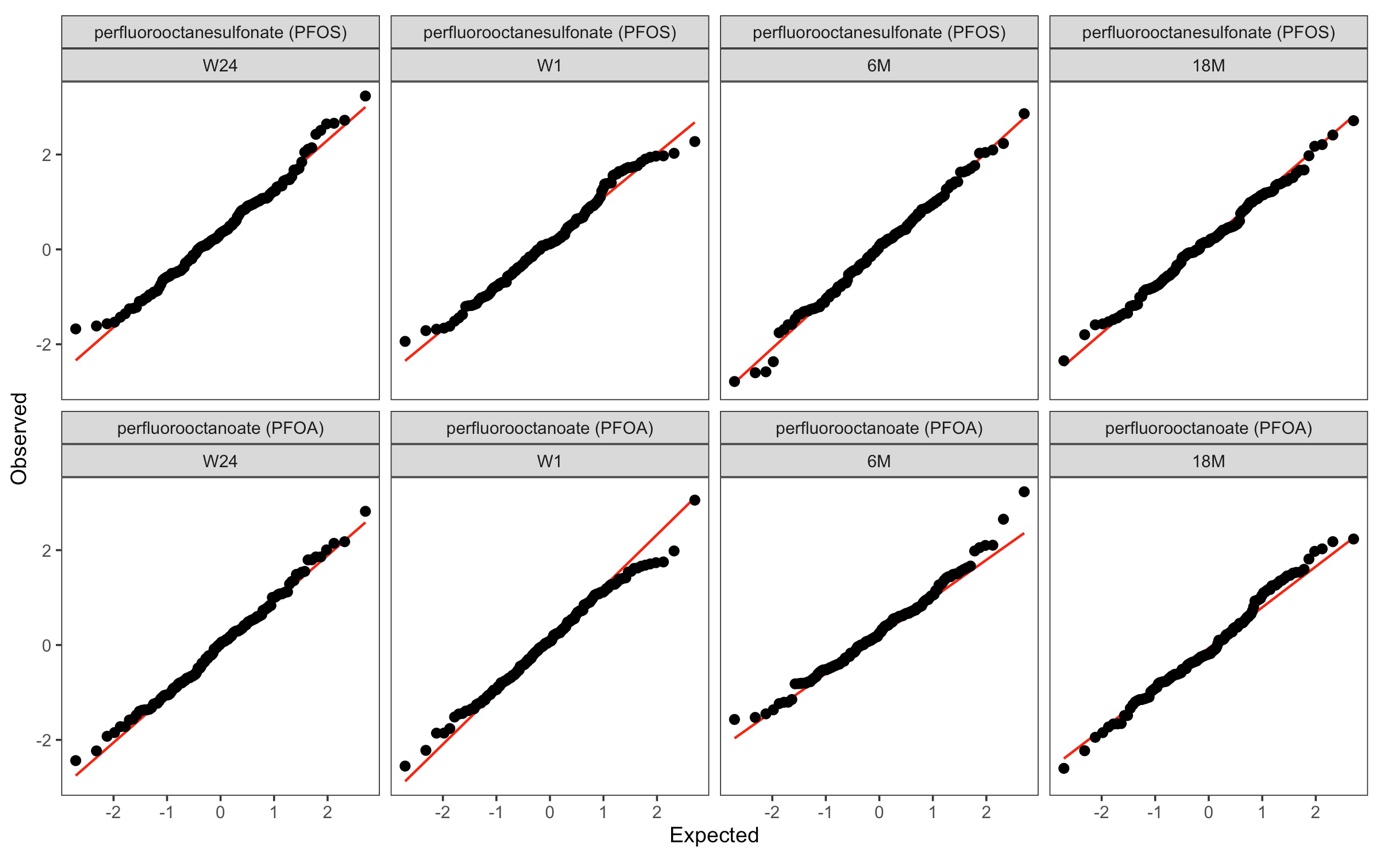


**Figure E4**

Epigenome-wide association (t-statistic) between child PFOA/PFOS concentrations and methylation levels at age 6 show no inflation.

a) QQ plot for p-values of the maternal week 24 PFOS analysis b) QQ plot for p-values of the maternal week 24 PFOA analysis c) QQ plot for p-values of the maternal week 1 PFOS analysis d) QQ plot for p-values of the maternal week 1 PFOA analysis e) QQ plot for p-values of the child 6 months PFOS analysis f) QQ plot for p-values of the child 6 months PFOA analysis g) QQ plot for p-values of the child 18 months PFOS analysis h) QQ plot for p-values of the child 18 months PFOA analysis i) QQ plot for p-values of the child 6 years PFOS analysis j) QQ plot for p-values of the child 6 years PFOA analysis. (GIF: genomic inflation factor)


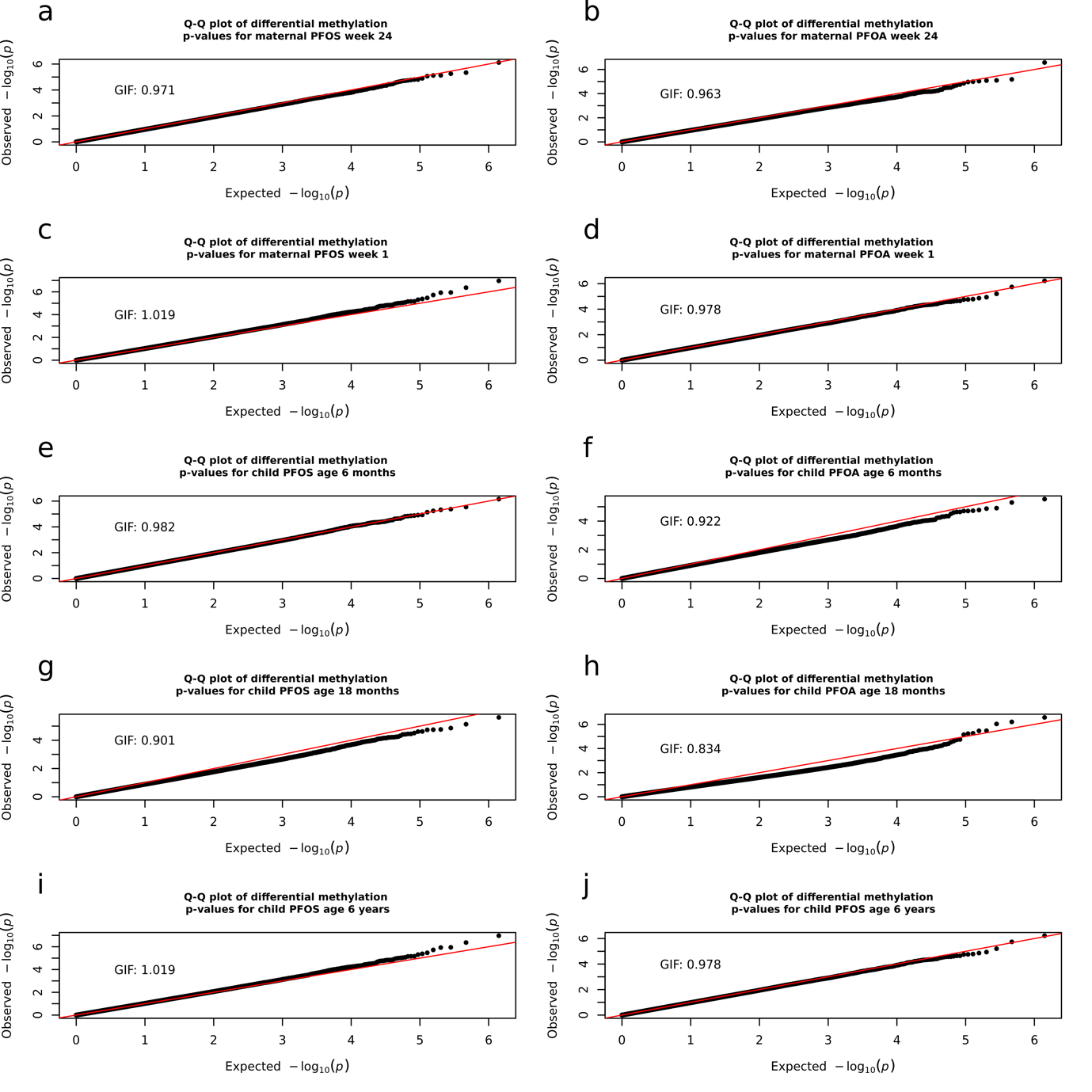

Supplement: Supplementary Figs — . [file mmc2.docx]
